# Childhood paternal abuse and low paternal affection predict adult panic symptoms 18 years later via actigraphy-indexed sleep disruptions

**DOI:** 10.1017/S0033291726104577

**Published:** 2026-06-01

**Authors:** Nur Hani Zainal, Natalia Van Doren

**Affiliations:** 1 National University of Singapore, Singapore; 2 Yeo Boon Khim Mind-Science Centre, Singapore; 3 UCSF: University of California San Francisco, USA

**Keywords:** actigraphy, child maltreatment, panic disorder, parental affection, sleep disturbance

## Abstract

**Background:**

Child abuse and low parental affection are established risk factors for higher adulthood panic disorder (PD) severity, but their plausible sleep mediators are under-investigated. We thus examined how actigraphy indices mediated the links between child parental abuse and affection deficits to adulthood PD severity.

**Methods:**

Community-dwelling adult participants (*N* = 1,054) completed a series of self-reports on child parental abuse and affection at Wave 1. An eight-day actigraphy protocol was conducted at Wave 2, 9 years later. Telephone-administered clinical interviews assessing PD symptoms were conducted in W1 and Wave 3, separated by 18 years. A series of bias-corrected, bootstrapped causal mediation analyses was performed.

**Results:**

Paternal, but not maternal abuse, predicted higher rest- and sleep-stage actigraphy markers after 9 years (βs = 0.263–469.79, *p* < .001), comprising more activity counts, longer wake time, higher wake time percentage, and wake bouts. These markers, thereby, mediated the trajectory from paternal abuse to higher PD severity (average causal mediation effects (ACMEs): βs = 0.003–0.020, 95% CIs excluding zero, *p* < .001). Likewise, paternal affection deficits predicted greater disturbances in rest- and sleep-stage actigraphy (all *p* < .05), thereby mediating the link to greater PD severity (ACMEs: βs = 0.001–0.002, *p* ≤ .04). Neither maternal abuse nor affection was a significant mediator.

**Discussion:**

These outcomes aligned with, but do not verify, a causal mediation argument wherein actigraphy-derived nocturnal sleep–wake disturbances partially accounted for the trajectory from adverse paternal caregiving encounters to adulthood PD severity. Strategically targeting sleep disturbances may reflect a viable intervention approach for persons with past child paternal abuse learning histories.

## Introduction

Panic disorder (PD) is a common and serious anxiety disorder. It is marked by repeated, unanticipated panic attacks, chronic worry, and avoidance patterns (American Psychiatric Association, 2013). Epidemiological studies have shown that PD affects about 2%–3% of adults globally. Lifetime prevalence in community samples ranges from 3% to 5% (de Jonge et al., 2016; Kessler et al., 2006). The condition places a considerable burden on individuals and society. This is due to increased healthcare utilization, work dysfunction, and comorbidities such as more symptoms of major depressive disorder (MDD) and generalized anxiety disorder (GAD; Hirschfeld, 2001; Kim, Kim, & Lee, 2021). Empirically supported cognitive-behavioral therapy (Cuijpers et al., 2025; Papola et al., 2023) and pharmacotherapy (Batelaan et al., 2017) are available. However, many people with PD face chronic recurrent symptoms throughout adulthood. Identifying distal risk factors for PD symptoms is, therefore, an essential public health consideration. This helps inform early detection and prevention strategies.

Developmental psychopathology models propose that early parenting environments play a key role in risk for adult anxiety disorders. The attachment model asserts that parent–child interactions shape emotion regulation, internal models of safety, and the body’s stress response (Huang & Liu, 2025; Mandelli, Petrelli, & Serretti, 2015). These theories posit that parental abuse and lack of affection blunt emotion regulation and weaken secure attachment (Warmingham et al., 2023). They also posit that these factors sensitize children to perceived threats and increase fear responses (Zoladz et al., 2022). These reactions can contribute to the development and persistence of PD symptoms. The hyperarousal model adds that ongoing negative parenting chronically over-activates the body’s fight-flight-freeze system (McLaughlin, Sheridan, Alves, & Mendes, 2014). Persistent hyperarousal raises the risk for long-term panic symptoms.

Consistent with these propositions, prior research has shown that childhood emotional and physical abuse are substantially linked to heightened PD symptoms and diagnosis across the lifespan (Goodwin, Fergusson, & Horwood, 2005; Kim et al., 2023). Empirical data have also demonstrated that a lack of parental affection, care, and warmth – separate from abuse – predicts higher symptom severity of anxiety disorders (Boullion, Linde-Krieger, Doan, & Yates, 2023). Previous studies have emphasized that paternal behaviors, particularly paternal sensitivity, are key predictors of secure attachment patterns (Brown, Mangelsdorf, & Neff, 2012). A longitudinal study found that positive reappraisal mediated the link between baseline parental abuse and affection deficits and later increased GAD severity (Ng, Zainal, & Newman, 2024). Together, this body of work implies that cognitive-affective processes may mediate the influence of adverse paternal caregiving on PD symptoms. However, the mediators through which childhood parental abuse and affection deficits may lead to long-term PD symptoms remain open to inquiry.

Sleep disturbances may mediate the impact of early parental abuse and lack of affection on adult PD symptoms. Hyperarousal frameworks suggest that persistent threat exposure in formative years increases the sensitivity of the sympathetic autonomic nervous system (ANS). This leads to heightened nocturnal arousal and disrupted restorative sleep (Pfaff, Jud, & Schlarb, 2021). Dysregulation of the hypothalamic–pituitary–adrenal (HPA) axis, which is linked to child abuse (Murphy et al., 2022), may also cause fragmented sleep patterns and increased nighttime physical activity.

Aligned with these frameworks, systematic reviews have documented broad links between child abuse experiences and sleep disturbances during youth (Schonning, Sivertsen, Hysing, Dovran, & Askeland, Schonning et al., 2022) and adult development (Semsar, Mousavi, Tran, & Kuhlman, 2021). Longitudinal cohort studies further showed that parental child maltreatment could predict sleep disorders in adulthood, such as daytime dysfunction, insomnia, and hypersomnia (Greenfield, Lee, Friedman, & Springer, 2011; Kalantar‐Hormozi & Mohammadkhani, 2024). Sleep disturbances have also reliably predicted future anxiety symptoms. Prolonged wakefulness and sleep fragmentation could magnify interoceptive threat appraisal and stress reactivity, which are critical to maintaining the panic cycle (Babson, Feldner, Trainor, & Smith, 2009). Child abuse is particularly linked to somatic arousal mediators, which may forecast clinical sleep disturbances later in adulthood (Reffi et al., 2022). This supports a mediational proposition. Collectively, these data support the hypothesis that objectively measured sleep disturbances could mediate the effects of childhood parental adversity on long-term PD risk.

However, the literature has notable gaps that the current study aimed to circumvent. Most existing studies have relied on either self-report measures of sleep quality or one-night polysomnography protocols to examine sleep disturbances in this setting (Pfaff, Jud, & Schlarb, 2021). Yet, self-reports may be subject to recall biases and subjective misperceptions of sleep, and while polysomnography is the gold standard, it is costly and limited to single-night assessments in laboratory settings (Lehrer et al., 2022). In contrast, actigraphy offers a cost-effective, ecologically valid approach, passively recording sleep–wake trends across several consecutive days in natural habitats, using a wrist-worn sensor (Blackwell et al., 2008). Actigraphy has also shown convergent validity with polysomnography for markers such as fragmented sleep and wake after sleep onset (WASO; Saleh et al., 2025). Additionally, the American Academy of Sleep Medicine supports the use of actigraphy for assessing circadian sleep–wake rhythms (Smith et al., 2018). When paired with sleep diaries, actigraphy provides optimal measures of sleep and rest across days, yielding diurnal sleep–wake data not captured by single-night polysomnography (Campanini et al., 2017). Another limitation identified in the literature is that optimal mediation research necessitates at least three assessment waves to temporally sequence the predictor, mediator, and outcome in a logical mediational chain (Maxwell & Cole, 2007). Furthermore, maternal and paternal caregiving experiences may confer differential effects on adult PD symptoms due to varying interaction frequencies and attachment patterns (Moller, Nikolic, Majdandzic, & Bogels, 2016). To address these limitations, the present study used a three-wave dataset to test the mediational value of actigraphy in the link from child parental abuse and affection deficits to future PD symptoms.

Accordingly, the current study evaluated whether actigraphy-derived sleep disturbance markers – assessed during rest, sleep, and active wake stages – served as mediators linking childhood parental abuse or affection deficits (measured during childhood) to PD severity measured 18 years later. Specifically, we tested whether childhood parental abuse predicted greater sleep disturbances at a 9-year follow-up, which would then predict higher PD severity after a further 9 years, thus clarifying the indirect path from abuse to adult PD severity. Similarly, we tested whether lower parental affection was associated with greater subsequent sleep disturbances, which would mediate the association between affection deficits and later PD severity. These models were tested using bias-corrected mediation tests in a community-dwelling adult cohort from the Midlife Development in the United States (MIDUS) study, statistically controlling for baseline PD severity and key covariates. Consistent with our objective assessment approach, we anticipated that sleep and rest disturbances (e.g. fragmented sleep or nighttime wakefulness) would mediate these links more strongly than disturbances during active wake stages.

## Method

### Participants and study design

Community-dwelling adults (*N* = 1,054) participated in the MIDUS at Wave 1 (W1; 1995–1996; Brim et al., 2020), Wave 2 (W2; 2004–2006; Ryff et al., 2021), and Wave 3 (W3; 2013–2014; Ryff et al., 2019). The final analytic sample was selected because it provided sufficient data across all assessment waves to answer the research questions. Specifically, these participants underwent the actigraphy protocol at W2, elaborated on in the following sections (Teas & Friedman, 2021). At W1, their mean age was 46.19 (*SD* = 11.81, range = 25–74). There were 577 women (54.70%) and 477 men (45.30%). Regarding education, 464 (44.00%) received a college education or higher (Table 1 and Online Supplemental Materials [OSM] Table S1).

**Table 1.** Descriptive statistics of key study variables

Continuous variables	*M*	(*SD*)	Minimum	Maximum	Skewness	Kurtosis
W1 Age (years)	46.19	(11.81)	25.00	74.00	0.30	−0.68
W1 Child maternal abuse	4.69	(1.99)	3.00	12.00	1.54	2.36
W1 Child paternal abuse	5.03	(2.15)	3.00	12.00	1.35	1.57
W1 Child maternal affection	18.94	(4.95)	6.00	54.00	1.41	12.99
W1 Child paternal affection	17.71	(8.82)	6.00	54.00	2.65	8.97
W1 Panic disorder severity	0.55	(1.33)	0.00	6.00	2.42	4.81
W3 Panic disorder severity	0.44	(1.10)	0.00	6.00	2.95	8.68
W1 Household income	79863.73	(63896.23)	0.00	300000.00	1.41	1.97
W1 Generalized anxiety disorder severity	12.61	(7.32)	8.00	32.00	1.12	−0.40
W1 Major depressive disorder severity	0.88	(2.14)	0.00	9.00	2.19	3.22
W1 Parental psychopathology	0.62	(0.78)	0.00	4.00	1.65	3.03

Categorical variables	*n*	(%)				
Sex						
Male	477	(45.30)				
Female	577	(54.70)				
Education level						
College education and above	464	(44.00)				
High school	238	(22.60)				
No high school degree	51	(4.84)				
Some college	300	(28.50)				
Declined to disclose	1	(0.09)				
Race						
Multiracial	8	(0.76)				
White	961	(91.20)				
African American	29	(2.75)				
Native American or Pacific Islander	5	(0.47)				
Asian	2	(0.19)				
Others	19	(1.80)				
Declined to disclose	30	(2.85)				

*Note*: W1, wave 1 (1995–1996); W2, wave 2 (2004–2006); W3, wave 3 (2013–2014). Refer to Supplementary Materials Table S1 in the online supplemental materials (OSM) for more details on the W2 actigraphy mediators.

### Procedures

Participants completed a clinical interview and the Composite International Diagnostic Interview-Short Form (CIDI-SF; Kessler et al., 1998) to assess PD severity at W1 and W3. At W1, they also completed a demographic questionnaire. At W2, they followed an 8-day actigraphy protocol to track physical activity and sleep patterns, starting Tuesday morning and ending the following Tuesday morning after a MIDUS site visit to standardize the protocol (Love, Seeman, Weinstein, & Ryff, 2010; Teas & Friedman, 2021).

### Measures

**W1 Childhood Parental Abuse.** Retrospective child maternal and paternal abuse was measured using the Revised Conflict Tactics Scale (Straus, 1979). For each parental figure, one item assessed emotional abuse (e.g. ‘did or said something to spite you’), one assessed physical abuse (e.g. ‘threw something at you’), and one assessed severe physical abuse (e.g. ‘burned or scalded you’). In nontraditional families, participants referred to their closest maternal and paternal figures. Abuse severity was rated on a 4-point Likert scale (1 = *never* to 4 = *often*). Maternal and paternal abuse rates ranged from 75.5% versus 70.6% (*never*); 18.7% versus 21.3% (*rarely*); 4.5% versus 5.9% (*sometimes*); and 1.3% versus 2.2% (*often*). The measure demonstrated good internal consistency (α = .82 for maternal abuse, α = .84 for paternal abuse herein), good retest reliability, and strong construct validity (Ng, Zainal, & Newman, 2024; Straus, Hamby, Boney-McCoy, & Sugarman, 1996).

**W1 Childhood Parental Affection.** Retrospective child, maternal, and paternal affection were assessed using Rossi’s (2001) scale. For each parental figure, participants self-reported their level of empathic understanding of the individual’s problems and worries, as well as how well the parental figure listened, expressed love, and provided attention when needed. Respondents rated six items per parent on a 4-point Likert scale (1 = *never* to 4 = *often*). Affection rates for mothers and fathers were: 7.8% versus 17.7% (*never*), 25.2% versus 37.0% (*rarely*), 56.7% versus 37.0% (*sometimes*), and 10.3% versus 8.3% (*often*). The scale showed high internal consistency (α = .93 for maternal, α = .98 for paternal), strong retest reliability, and solid construct validity, as supported by previous nonpsychometric studies (Ng, Zainal, & Newman, 2024; Rossi, 2001; Ryff & Singer, 2004).

#### W1 and W3 PD Severity

The MIDUS research team used telephone clinical interviews and administered the CIDI-SF to assess PD diagnosis and severity in the past 12 months (Kessler et al., 1998). Each participant reported PD symptoms. Symptoms were marked as ‘0’ for *absent* and ‘1’ for *present*, and these pertained to both cued and noncued panic attacks. The recorded symptoms were chills/hot flashes, discomfort/pain/tightness in the chest or stomach, heart palpitations, sweating, trembling/shaking, and a sense of unreality in the surroundings (Gigantesco & Morosini, 2008). PD severity was calculated as the sum of these PD symptoms, ranging from 0 (*lowest severity*) to 6 (*highest severity*). The CIDI-SF PD severity scale (total sum score) has demonstrated good internal consistency (α = .86 at both W1 and W3 herein). Prior nonpsychometric research also found that this measure has good convergent validity and strong discriminant validity (Michal et al., 2025; Zainal, Soh, & Van Doren, 2024).

#### W2 physical activity and sleep actigraphy indicators

An actigraphy wearable passively captured physical activity and sleep variables in three distinct stages: resting (a period of stillness prior to actual sleep onset), sleeping (periods identified when the participant is asleep), and active wake (periods of physical activity and alertness after waking) (Chung, 2017). Seven indicators were recorded during all stages: activity counts (total, average, and maximum), wake time length, percentage of wake time, wake bouts, and average sleep bouts. Four additional sleep-specific variables included sleep onset latency (SOL), time spent dozing before rising, sleep efficiency, and WASO (Owens et al., 2017). Data were collected in 30-second to 1-minute intervals. Participants also used diaries to record their sleep experiences, marking the start and end of resting stages (Kim et al., 2016; Mezick, Wing, & McCaffery, 2014).

Actigraphy measures sleep–wake rhythms by tracking movement (Smith et al., 2018). Sleep and wake bouts count the number of uninterrupted sleep or awake periods in a day (Di Marco et al., 2023). More wake bouts lead to fragmented sleep and increased nighttime movement (Saleh et al., 2025). Sleep efficiency is the percentage of time spent asleep while in bed; low scores indicate greater wakefulness (Reed & Sacco, 2016). SOL is how long it takes to fall asleep (Fekedulegn et al., 2020). WASO is the total wake time after falling asleep, but before final awakening (Nakazaki et al., 2014). Activity counts reflect movement detected during set intervals, with higher counts suggesting restlessness (Tsanas, Woodward, & Ehlers, 2020).

### Data analyses

**Figure 1.**
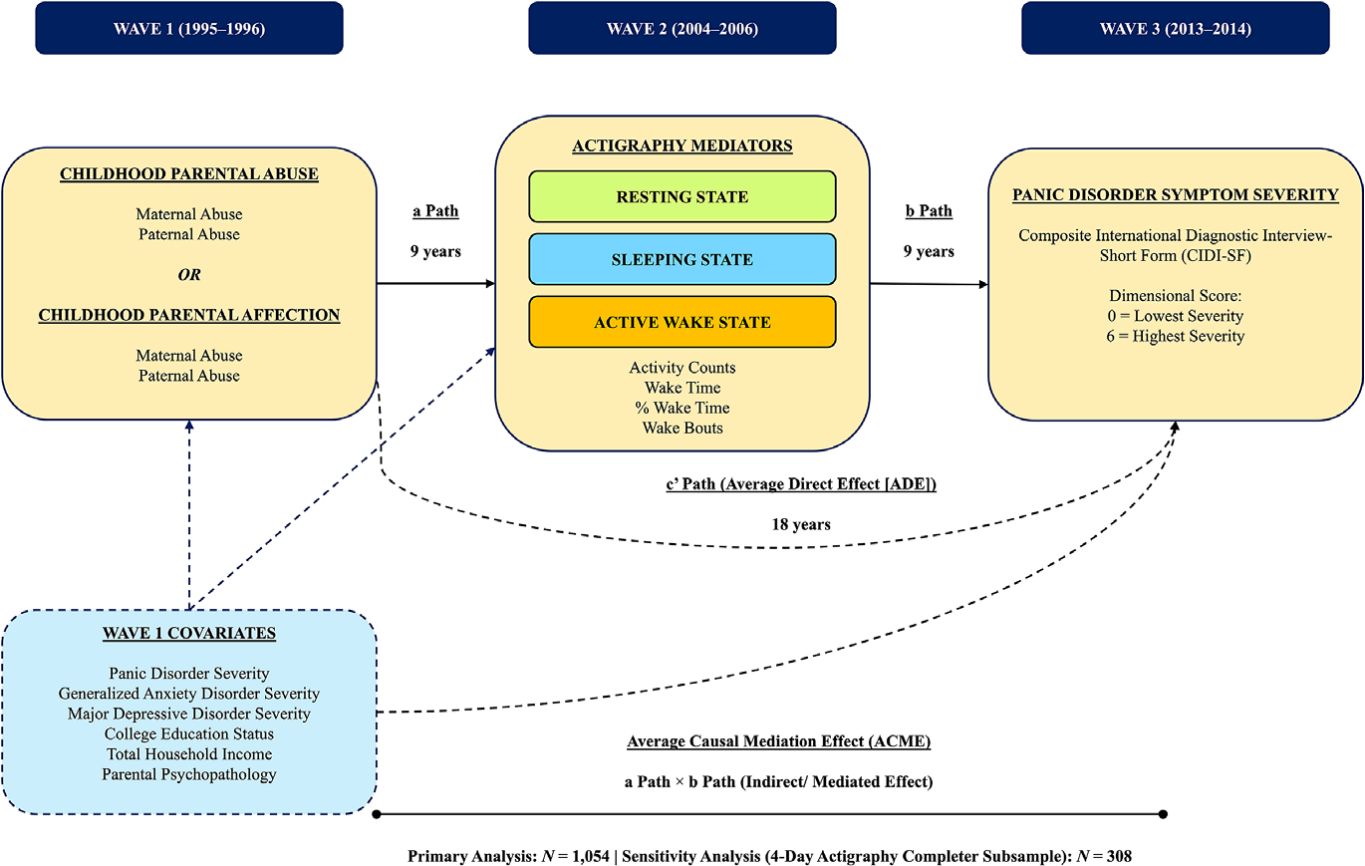
Schematic overview of mediational pathways examined in the present study. *Note:* CIDI-SF, ‘Composite international diagnostic interview-short form’; ACME, ‘average causal mediation effect’; (a) Path, predictor–mediator pathway; (b) Path, mediator–outcome; ADE, ‘average direct effect’.

Figure 1 shows a schematic overview of the study’s data analytic plan. *R* software (version 4.4.2; R Core Team, 2024) was used for all data preprocessing and analyses. Several preprocessing steps were conducted before the mediational analyses. First, missing data accounted for 41% of the dataset. Such high levels of missingness are expected in actigraphy research. This is due to incomplete event-index data, long nonwear periods, and technical issues (Slyepchenko et al., 2023). To reduce potential bias, random forest imputation (Mayer, 2024) was used. This method handles missing-not-at-random (MNAR) patterns and can accommodate complex interactions and nonlinearities. It is more effective than standard multiple imputation (Shah et al., 2014; Tang & Ishwaran, 2017). Second, regression assumption checks showed that the variables were suitable for mediation analyses. These checks covered linearity, independence of errors, homoscedasticity, normality, multicollinearity, and influential observations.

A series of causal mediation analyses (Imai, Keele, & Tingley, 2010; Imai, Keele, Tingley, & Yamamoto, 2011) was conducted. These examined how specific actigraphy variables mediated the link between child abuse and 18-year PD severity. The actigraphy variables covered resting, sleeping, and active wake stages. Causal mediation was used because it provides nonparametric estimates under less stringent functional-form and distributional assumptions than conventional models (VanderWeele & Vansteelandt, 2014). This is important given the nonlinear, skewed actigraphy data and the variety of abuse and affection exposures. The method also enables formal identification of contexts, allows extensive covariate adjustment, and provides robust estimates via quasi-Bayesian simulations (Tingley et al., 2014). Such simulations are less straightforward to implement in standard structural equation models. Step 1 examined the ‘a path,’ the relationship between the predictor (maternal or paternal abuse or affection at W1) and the mediator (a specific actigraphy variable at W2). Step 1 included either both maternal and paternal abuse or both maternal and paternal affection. Step 2 investigated the ‘b path,’ the association between the mediator and the outcome (W3 PD severity). All analyses controlled for W1 PD severity to account for its confounding effect. We used the mediation package (Tingley et al., 2014), which employed a quasi-Bayesian method. This estimated the average causal mediation effect (ACME), the total effect, the average direct effect (ADE), and the percentage of variance in the association between the predictor and outcome explained by the mediator (Imai & Yamamoto, 2017). The ACME refers to the average indirect causal effect. The ADE is the effect that occurs not through the mediator. In the Results section, we focused on reporting the ‘a paths’ and ACME, as they addressed Hypotheses 1 and 2 directly.

Each model was entered separately, with either maternal or paternal indicators (abuse or affection) as the key predictor. A W2 actigraphy index was a possible mediator of W3 PD severity. Stated differently, we did not enter maternal and paternal markers together in a single mediation model. Each was evaluated in a separate model to isolate its distinct mediation effects. All analyses included the full sample (*N* = 1,054). They used the maximum relevant data across all assessment waves (see OSM for details). Beyond W1 PD severity, the model adjusted for other potential confounders. These included education, household income (socio-economic status [SES] markers), parental psychopathology, MDD severity, and GAD severity. These confounders were selected because they might contribute to the mediational links of interest (Greene, Haisley, Wallace, & Ford, 2020; Meuret, Kroll, & Ritz, 2017; Sareen, Afifi, McMillan, & Asmundson, 2011), ultimately leading to the W3 PD severity outcome.

Finally, sensitivity analyses were conducted with participants who completed at least the first 4 days of actigraphy (*N* = 308). The threshold for statistical significance in all mediation analyses was set at *p* < .05 to maximize statistical power and reduce the risk of Type II error. Another set of sensitivity analyses assessed the statistical robustness of significant ACME estimates to potential violations of the sequential ignorability assumption (i.e. ρ-based sensitivity analyses). These used the *medsens*() function in the *mediation* package (Imai, Tingley, & Yamamoto, 2013; Tingley et al., 2014).

## Results

### W1 childhood parental abuse as a predictor of W3 PD severity via W2 actigraphy

Partially consistent with Hypothesis 1, higher childhood paternal, but not maternal, abuse significantly predicted actigraphy markers across rest and sleep stages after 9 years, thereby significantly predicting greater PD severity. During resting stages, paternal abuse significantly predicted more total activity counts (β *=* 469.79, *SE* = 100.83), mean activity counts per minute (β *=* 0.914, *SE* = 0.204), maximum activity counts (β *=* 5.25, *SE* = 2.11), longer wake time (β *=* 1.93, *SE* = 0.382), higher percentage of wake time (β *=* 0.353, *SE* = 0.075), and counts of wake bouts (β *=* 0.562, *SE* = 0.140; all *p* < .001; Supplementary Materials Table S2). Within sleeping stages, these actigraphy markers significantly mediated the path from paternal abuse to future PD severity: more total activity counts (β = 270.60, *SE* = 57.03), mean activity counts per minute (β = 0.595, *SE* = 0.129), longer wake time (β = 1.243, *SE* = 0.267), higher percentage of wake time (β = 0.263, *SE* = 0.059), and counts of wake bouts (β = 0.472, *SE* = 0.120; all *p* < .001; Supplementary Materials Table S3). However, active wake actigraphy indices did not significantly mediate the link from maternal or paternal abuse to PD severity (Supplementary Materials Table S3).

**Figure 2.**
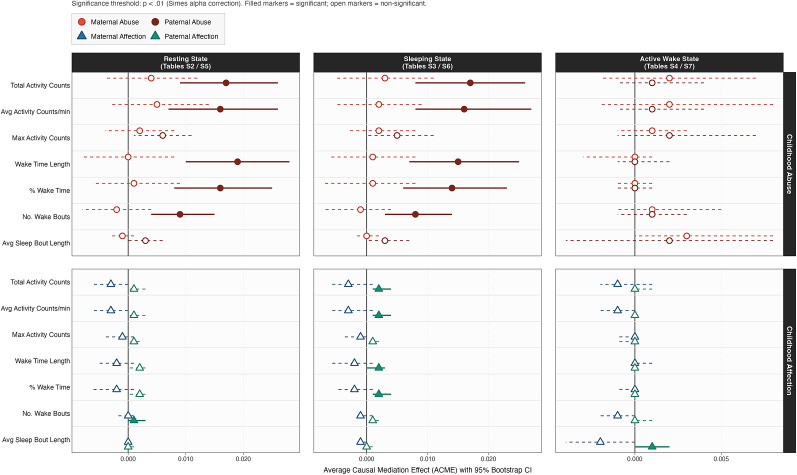
ACME Forest Plots Across Actigraphy States and Childhood Parental Abuse or Affection *(N = 1,054)*. *Note:* ACME, ‘average causal mediation effect’; Circles, Abuse predictors (online supplemental materials [OSM] Supplementary Materials Tables S2–S4); Triangles, Affection predictors (OSM Supplementary Materials Tables S5–S7). Points indicate ACME point estimates, and bars represent the 95% confidence intervals (CIs). Darker color fill represents paternal predictors. Lighter color fill indicates maternal predictors. All analyses adjusted for these Wave 1 covariates: panic disorder severity; generalized anxiety disorder severity; major depressive disorder severity; college education status; total household income; and parental psychopathology status.

Simultaneously, significant ACMEs in the resting stages were found in the links from paternal abuse to PD severity via these markers (Figure 2; Supplementary Materials Table S2): total activity counts (β = 0.020, 95% CI [0.010, 0.030]), mean activity counts per minute (β = 0.016, 95% CI [0.007, 0.026]), maximum activity counts (β = 0.006, 95% CI [0.001, 0.011]), wake time length (β = 0.019, 95% CI [0.010, 0.028]), percentage of wake time (β = 0.016, 95% CI [0.008, 0.025]), and counts of wake bouts (β = 0.009, 95% CI [0.004, 0.015]; all *p* < .001). Significant ACMEs in the sleeping stages were replicated from paternal abuse to PD severity via these indices (Supplementary Materials Table S3): total activity counts (β = 0.017, 95% CI [0.008, 0.026]), mean activity counts per minute (β = 0.016, 95% CI [0.008, 0.027]), maximum activity counts (β = 0.005, 95% CI [0.000, 0.011]), wake time length (β = 0.015, 95% CI [0.007, 0.025]), percentage of wake time (β = 0.014, 95% CI [0.006, 0.023]), counts of wake bouts (β = 0.008, 95% CI [0.003, 0.014]), and mean sleep bouts (β = 0.003, 95% CI [0.001, 0.007]). However, no ACME estimates were significant when maternal abuse was the predictor, and for active wake stage indices (Supplementary Materials Tables S2–S4).

### W1 childhood parental affection as a predictor of W3 PD severity via W2 actigraphy

Partially consistent with Hypothesis 2, lower childhood paternal, but not maternal, affection significantly predicted actigraphy markers across rest and sleep stages after 9 years, thereby predicting greater PD severity (Figure 2; Supplementary Materials Table S5). During resting stages, these actigraphy indices significantly mediated the link from lower paternal affection to higher PD severity: higher total activity counts (β = 0.001, 95% CI [0.000, 0.003], *p* = .040), mean activity counts per minute (β = 0.001, 95% CI [0.001, 0.003], *p* = .040), longer wake time (β = 0.002, 95% CI [0.001, 0.003], *p* = .012), percentage of wake time (β = 0.002, 95% CI [0.001, 0.003], *p* = .024), and counts of wake bouts (β = 0.001, 95% CI [0.001, 0.003], *p* = .008). Similarly, during sleeping stages, these actigraphy markers significantly mediated the link between lower paternal affection and higher PD severity (Figure 2; Supplementary Materials Table S6): higher total activity counts (β = 0.002, 95% CI [0.001, 0.004], *p* = .008), mean activity counts per minute (β = 0.002, 95% CI [0.001, 0.004], *p* = .004), longer wake time (β = 0.002, 95% CI [0.000, 0.003], *p* = .008), percentage of wake time (β = 0.002, 95% CI [0.001, 0.004], *p* = .004), and counts of wake bouts (β = 0.001, 95% CI [0.000, 0.002], *p* = .016). During the active wake stage, the only significant mediation outcome was the effect of lower paternal affection on higher PD severity through mean sleep bouts (β = 0.001, 95% CI [0.000, 0.002], *p* = .004; Figure 2; Supplementary Materials Table S7). However, maternal affection as a predictor did not yield any significant ACME estimates.

### Complete-case sensitivity analyses

**Figure 3.**
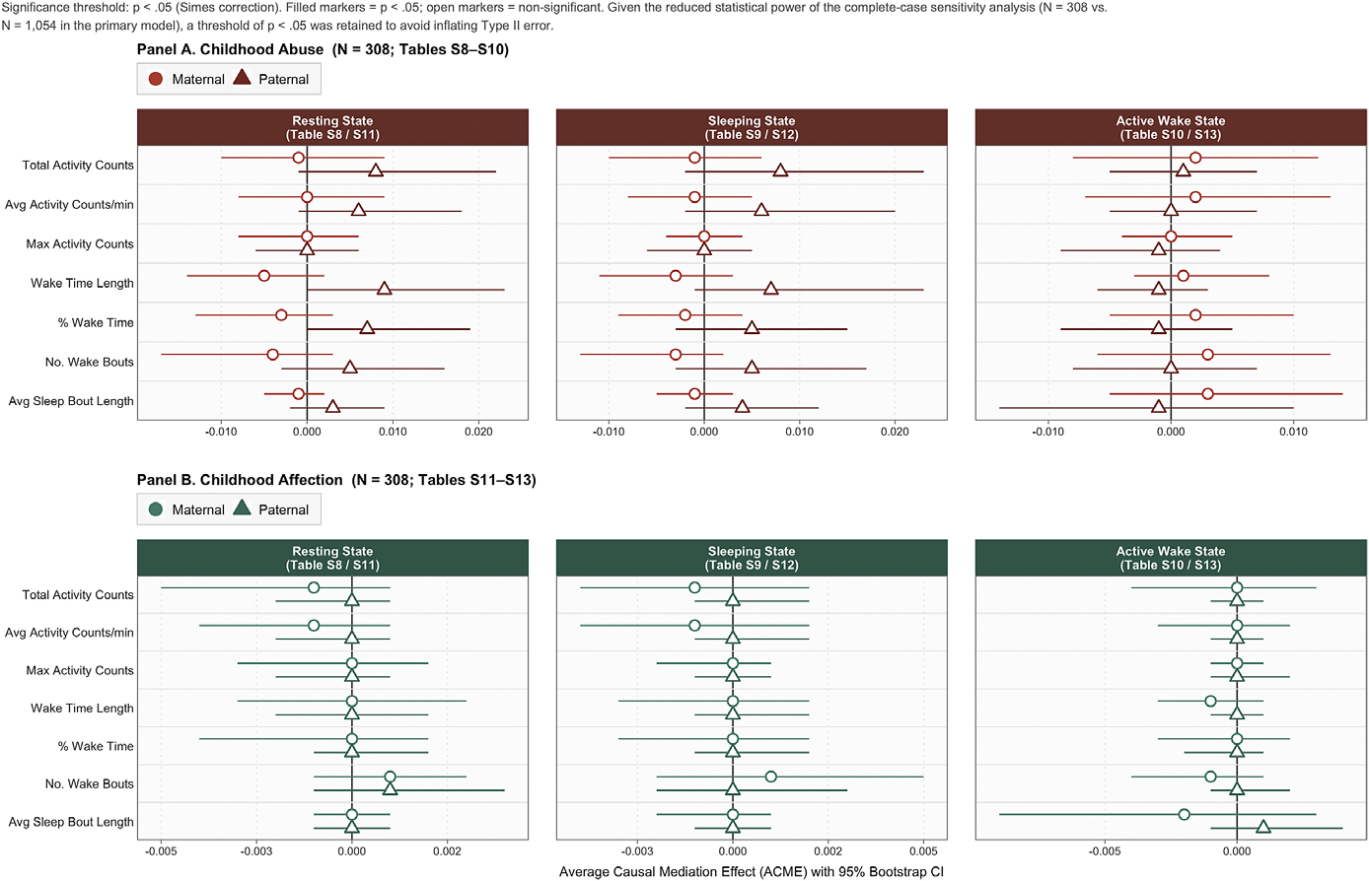
Sensitivity analyses of ACME forest plots across actigraphy states and childhood parental abuse or affection (N = 308). *Note:* ACME, ‘average causal mediation effect’; Circles, Abuse predictors (online supplemental materials [OSM] Supplementary Materials Tables S8–S10); Triangles, Affection predictors (OSM Supplementary Materials Tables S11–S13). Points indicate ACME point estimates, and bars represent the 95% confidence intervals (CIs). All analyses adjusted for these Wave 1 covariates: panic disorder severity; generalized anxiety disorder severity; major depressive disorder severity; college education status; total household income; and parental psychopathology status.

Despite the expected attenuation in the number of statistically significant findings in the sensitivity analyses, given the smaller sample size and reduced power, the directions of effects remained similar in this series of complete-case analyses (Figure 3; Supplementary Materials Tables S8–S13). Longer resting state wake length still significantly mediated the link from higher paternal abuse to greater PD severity (β = 0.009, 95% CI [0.001, 0.023], *p* = .040). This directional consistency across all point estimates, despite fewer statistically significant outcomes, indicates robustness in the primary results rather than systematic bias.

### ρ-Based sensitivity analyses for unmeasured confounding

The ρ parameter estimates the residual association between the mediator and the outcome residual terms. Under the sequential ignorability condition, ρ = 0. The key value ρ* indicates the strength of ρ at which the ACME becomes statistically nonsignificant; thus, a lower absolute ρ* suggests more sensitivity to unmeasured confounding, and a higher value reflects greater robustness. For significant paternal abuse mediation in actigraphy rest and sleep stages, ρ* values were between 0.0530 and 0.2160 (Supplementary Materials Table S14). For significant paternal affection mediation, rest- and sleep-stage ρ* values were between 0.0520 and 0.2160 (Supplementary Materials Table S15). Neither maternal abuse nor affection models yielded significant mediation effects (Supplementary Materials Tables S14 and S15). Together, these values denote weak-to-moderate robustness to unmeasured confounding.

## Discussion

Our three-wave, 18-year prospective study supports a mediational account. Adverse childhood parental experiences were linked to higher PD severity in adulthood. This link occurred through actigraphy-based sleep–wake disturbances. Higher childhood paternal–rather than maternal–abuse coincided with more aberrant motor activity. There was more activity, higher wake-bout duration, and longer wake time during both rest and sleep stages about 9 years later. These sleep–wake markers were then linked to higher PD severity, also 9 years after the passive sensor measurements. Lower paternal affection showed a similar trend, with notable ACME estimates. These effects happened through rest- and sleep-stage activity counts, wake time percentage, wake bouts, and wake time length. All of these predicted future PD severity. Neither maternal abuse nor maternal affection mediated any effects on 18-year PD severity. The small ADE values across models indicated that learning histories with caregivers had minimal long-term impact on PD, beyond mediation by actigraphy. This highlights the importance of sleep–wake disturbances as a distal risk factor (Javakhishvili & Spatz Widom, 2021) and mediator (Semsar, Mousavi, Tran, & Kuhlman, 2021). The observational design and timing made it impossible to verify that unmeasured confounding was absent (Alvarez-Bartolo & MacKinnon, 2025). Therefore, these results are aligned with – but do not verify – a causal mediation sequence.

These results expand evidence linking adverse childhood experiences to adult sleep disturbances. Prior reviews showed broad links between childhood maltreatment and problematic sleep in adults and youths (Brown et al., 2022; Kajeepeta, Gelaye, Jackson, & Williams, 2015). However, neither review examined objective actigraphy markers tied to adult PD severity. We extended prior work using polysomnography and self-reported sleep. We identified actigraphy-based multiple-day indices as unique channels.

Paternal, but not maternal, childhood abuse was indirectly linked to adult sleep disturbance via actigraphy-indexed markers. One possible explanation is that fathers play a unique regulatory role in early sleep development, often promoting limit-setting and encouraging children to self-soothe (Ragni, De Stasio, & Barni, 2020; Tikotzky, Sadeh, & Glickman-Gavrieli, 2011). When a father is abusive, fear may replace support, preventing autonomous self-regulation and fostering chronic nighttime hyperarousal. This vigilance, developed in childhood, may persist into adulthood and lead to the fragmented sleep seen in our sample.

Second, paternal involvement in sleep caregiving is a consistently identified protective factor for sleep consolidation during infancy and early childhood. Studies link greater paternal engagement in bedtime routines to fewer night awakenings, longer sleep bouts, and easier settling to sleep (Ragni & De Stasio, 2020; Ragni, De Stasio, & Barni, 2020; Tikotzky, Sadeh, & Glickman-Gavrieli, 2011). Fathers are thought to buffer children against maternal stress and parenting adversity. They stabilize family functioning and indirectly support sleep consolidation (Ragni & De Stasio, 2020; Tikotzky, Sadeh, & Glickman-Gavrieli, 2011). When abuse is present, this dynamic shifts. The person expected to provide security becomes a source of threat. The child is left unsupported and faces fear and insecurity at night, losing the protective buffer. The resulting sleep disruption may be even more severe than paternal absence alone.

Paternal mind-mindedness, or viewing the child as having internal mental states, may also explain our findings. Actigraphy studies showed that higher paternal mind-mindedness at 18 months predicted better nighttime sleep consolidation later (Tetreault, Bernier, & Matte-Gagne, 2021). High-quality parent–child interactions were associated with sleep efficiency (Bordeleau, Bernier, & Carrier, 2012). Abuse disrupts this attunement, depriving youth and adults of the safety needed for healthy sleep development.

Taken together, these considerations suggest that paternal abuse may disrupt sleep through multiple, partially independent associations. These include the disruption of normative limit-setting functions, the neutralization of a key developmental protective buffer, the amplification of physiological stress reactivity, and the failure of attuned affective co-regulation. The differential pattern observed in the present study is consistent with theoretical accounts emphasizing the specific and non-redundant contributions of paternal caregiving to children’s sleep and stress system development (Ragni, De Stasio, & Barni, 2020). Our findings also aligned with studies showing that paternal parenting issues are often related more strongly to child internalizing problems than maternal behaviors in some contexts (Jami et al., 2020; Wittig & Rodriguez, 2019). Prior research found that paternal caregivers more often displayed coercive and controlling behaviors than maternal ones (DeGarmo, Nordahl, & Fabiano, 2016). Another study found that positive reappraisal mediated the link from paternal abuse and affection deficits to future GAD severity (Ng, Zainal, & Newman, 2024). Importantly, we found that sleep–wake dysregulation was a key, independent risk factor for PD. This expands our understanding of how adverse parental behaviors influence later PD symptoms.

The current findings could be explained by several theories. These remain conjectural due to our observational design and limited empirical tests. Mediation findings showed that rest- and sleep-stage actigraphy markers indicate fragmented sleep, more wakefulness, and more nocturnal motor activity. This supports the hyperarousal theory of anxiety-driven sleep problems (Kalmbach et al., 2018). Hyperarousal theory suggests that ongoing threat exposure sensitizes the sympathetic branch of the autonomic nervous system (Bonnet & Arand, 2010). It increases nighttime arousal, blocks restorative sleep, and sustains anxiety over time (Jenkins et al., 2024). The paternal-specific pattern could also reflect effects on the HPA axis (Gerritsen et al., 2017). Paternal abuse has been connected with HPA dysregulation across generations (Batchelor & Pang, 2019). This is likely due to the processes discussed earlier. High cortisol levels contribute to fragmented sleep and increased nighttime arousal (Carpenter et al., 2011). This magnifies PD symptoms over time.

Attachment theory may offer a complementary view. Paternal sensitivity is important for child attachment security (Olsavsky et al., 2020). Insecure attachment is linked to poorer sleep quality (Maunder, Hunter, & Lancee, 2011). Future studies should examine when paternal versus maternal affection supports physiological disengagement during rest and sleep.

Active wake-stage actigraphy markers in all models suggest disturbances happened mostly during the evening rest and sleep periods. This is instead of during the daytime, which would point to general hyperactivation. This contrast helps clarify the mediators. It reduces–but does not fully rule out–the possibility that actigraphy-based mediators reflect general hyperarousal rather than solely sleep-based issues (Smith et al., 2018). Future experimental and longitudinal studies are needed for firmer conclusions.

Several limitations should be considered. First, the observational design limits the strength of causal claims. We adjusted for PD, GAD, MDD symptoms, SES, and parental psychopathology. Still, residual confounding by unmeasured variables is possible. Second, retrospective parental abuse and affection reports may reflect reconstructed memories (Pinto Pereira, Rogers, & Power, 2021). Future studies should use longitudinal maltreatment measures and partner with social welfare agencies. Third, 41% data were missing. We applied random forest imputation to reduce MNAR bias (Tang & Ishwaran, 2017). In the complete-case analysis, many mediation effects lost significance due to less power. Only the paternal abuse–wake time mediation path stayed significant. This shows the need to replicate findings with larger samples and less missing data. Fourth, ACME effect sizes were small even when some predictor–mediator and mediator–outcome links were strong. These effects may be important in large samples predicting clinical outcomes. However, their clinical value should be interpreted cautiously and tested further in treatment studies. Relatedly, the ρ-based sensitivity analyses reflected weak-to-moderate robustness to unmeasured confounding effects across significant mediation links. This outcome suggested that unmeasured confounders might have either weakened or strengthened the estimated mediation effects. This underscores the importance of replication across experimental and longitudinal designs for a strong basis for causal inference. In addition, the PD assessment used DSM-III-R criteria because collection began in the 1990s. Given their strong overlap, replication efforts using DSM-5 measures would likely yield similar outcomes. Finally, the mostly White, college-educated MIDUS sample limits findings to similar groups.

This study has some strengths. We used actigraphy-based passive sensors, adding to prior self-report and polysomnography research. Our three-wave design suited mediation analysis. We also used a robust nonparametric mediation method to account for nonlinearities. Finally, we separately measured maternal and paternal caregiver effects to complement earlier research that combined both.

If replicated in diverse groups, these findings have clinical implications. Rest- and sleep-stage actigraphy markers could be key prevention targets for adults with childhood paternal abuse histories. These individuals may be at higher risk for future severe PD. Routine care could be improved by tracking actigraphy and validated self-reports of sleep among clients at risk. Residual sleep problems may keep PD symptoms going and require direct treatment. Evidence-based PD treatments using interoceptive exposure might benefit from the addition of cognitive-behavioral therapy (CBT) for insomnia (Jansson-Frojmark & Norell-Clarke, 2016). Attachment-based CBT approaches may also help (Newman, Castonguay, Jacobson, & Moore, 2015). Actigraphy data may improve care when combined with just-in-time adaptive interventions (JITAIs; Nahum-Shani & Murphy, 2026). JITAIs can warn clinicians about sleep declines when treating childhood abuse survivors at risk for PD (Zainal et al., 2025). The observed paternal-specific impacts–abuse and affection–highlight the importance of assessing paternal, alongside maternal, relationships during intake and for psychopathology understanding (Mestermann et al., 2023). Together, these strategies could enhance personalized case understanding and intervention plans.

## Supporting information

10.1017/S0033291726104577.sm001 Zainal and Van Doren supplementary material Zainal and Van Doren supplementary material

## Supplementary material

The supplementary material for this article can be found at http://doi.org/10.1017/S0033291726104577.
