# Supplementary material for: Childhood paternal abuse and low paternal affection predict adult panic symptoms 18 years later via actigraphy-indexed sleep disruptions
Source: Psychol Med. 2026 Jun 1;56:e170. doi: 10.1017/S0033291726104577 (PMC13234525; doi:10.1017/S0033291726104577)
Supplement: Zainal and Van Doren supplementary material [file S0033291726104577sup001.docx]

# Online Supplemental Materials (OSM)

## Data Analyses Details

The statistical significance threshold was set at *p* < .05, following an alpha correction and considering the sample size (Simes, 1986). To ease interpretation, Cohen’s *d* effect sizes were calculated with the formula *d* = 2*t*/√(*df*), where *t* represented the *t*-statistic of the unique parameter estimate, and *df* indicated the model degrees of freedom (Rosenthal, 1994). Given the clinical nature of the research aim and the large community sample, even *d* values of 0.1 can be practically significant (Lakens, 2013).

Table S1

*Descriptive Statistics of Key Study Variables*

| Continuous variables | *M* | (*SD*) | Minimum | Maximum | Skewness | Kurtosis |
| --- | --- | --- | --- | --- | --- | --- |
| W1 Age (years) | 46.19 | (11.81) | 25.00 | 74.00 | 0.30 | -0.68 |
| W1 Child maternal abuse | 4.69 | (1.99) | 3.00 | 12.00 | 1.54 | 2.36 |
| W1 Child paternal abuse | 5.03 | (2.15) | 3.00 | 12.00 | 1.35 | 1.57 |
| W1 Child maternal affection | 18.94 | (4.95) | 6.00 | 54.00 | 1.41 | 12.99 |
| W1 Child paternal affection | 17.71 | (8.82) | 6.00 | 54.00 | 2.65 | 8.97 |
| W1 PD severity | 0.55 | (1.33) | 0.00 | 6.00 | 2.42 | 4.81 |
| W3 PD severity | 0.44 | (1.10) | 0.00 | 6.00 | 2.95 | 8.68 |
| W1 Household income | 79863.73 | (63896.23) | 0.00 | 300000.00 | 1.41 | 1.97 |
| W1 GAD severity | 12.61 | (7.32) | 8.00 | 32.00 | 1.12 | -0.40 |
| W1 MDD severity | 0.88 | (2.14) | 0.00 | 9.00 | 2.19 | 3.22 |
| W1 Parental psychopathology | 0.62 | (0.78) | 0.00 | 4.00 | 1.65 | 3.03 |
| W2 Total activity counts (Resting) | 15509.50 | (6799.87) | 3594.00 | 53063.57 | 1.40 | 2.92 |
| W2 Average activity counts per minute (Resting) | 32.34 | (13.58) | 6.90 | 120.42 | 1.56 | 3.93 |
| W2 Maximum activity counts per minute (Resting) | 710.95 | (136.24) | 201.43 | 1363.29 | 0.36 | 1.85 |
| W2 Wake time length (Resting) | 75.56 | (25.54) | 23.14 | 234.07 | 1.06 | 2.35 |
| W2 Percentage of wake time (Resting) | 15.72 | (4.97) | 5.51 | 41.96 | 1.27 | 3.07 |
| W2 Wake bouts (Resting) | 42.71 | (8.93) | 10.71 | 95.71 | 0.00 | 3.12 |
| W2 Average sleep bouts (Resting) | 10.25 | (3.92) | 3.29 | 81.16 | 7.81 | 112.20 |
| W2 Total activity counts (Sleeping) | 9409.97 | (3799.66) | 1848.43 | 28696.57 | 0.98 | 1.75 |
| W2 Average activity counts per minute (Sleeping) | 21.21 | (8.57) | 4.82 | 74.10 | 1.23 | 2.85 |
| W2 Maximum activity counts per minute (Sleeping) | 583.69 | (121.57) | 185.43 | 1229.71 | -0.14 | 1.16 |
| W2 Wake time length (Sleeping) | 55.03 | (17.58) | 14.00 | 139.86 | 0.56 | 1.24 |
| W2 Percentage of wake time (Sleeping) | 12.41 | (3.90) | 3.77 | 31.08 | 0.88 | 2.11 |
| W2 Wake bouts (Sleeping) | 37.52 | (7.58) | 10.14 | 67.64 | -0.66 | 1.37 |
| W2 Average sleep bouts (Sleeping) | 11.25 | (4.36) | 4.26 | 81.44 | 6.47 | 76.04 |
| W2 Sleep onset latency (Sleeping) | 20.61 | (14.17) | 0.21 | 128.57 | 2.99 | 12.32 |
| W2 Wake after sleep offset (Sleeping) | 10.44 | (9.49) | 0.50 | 78.14 | 3.36 | 15.13 |
| W2 Sleep efficiency (Sleeping) | 81.09 | (6.17) | 44.27 | 93.61 | -1.59 | 4.93 |
| W2 Wake after sleep onset (Sleeping) | 55.04 | (17.60) | 14.00 | 139.86 | 0.56 | 1.24 |
| W2 Total activity counts (Active) | 339408.37 | (62616.52) | 62573.67 | 620944.17 | -0.17 | 4.49 |
| W2 Average activity counts per minute (Active) | 349.99 | (63.55) | 65.49 | 660.60 | -0.18 | 4.92 |
| W2 Maximum activity counts per minute (Active) | 1393.96 | (213.60) | 429.83 | 2406.83 | 0.22 | 3.87 |
| W2 Wake time length (Active) | 837.40 | (61.37) | 504.92 | 1085.75 | -1.73 | 6.11 |
| W2 Percentage of wake time (Active) | 87.01 | (5.72) | 48.62 | 97.64 | -2.59 | 8.29 |
| W2 Wake bouts (Active) | 54.37 | (18.35) | 7.33 | 158.17 | 2.04 | 6.54 |
| W2 Average sleep bouts (Active) | 2.30 | (0.82) | 1.22 | 19.29 | 9.91 | 179.72 |
| Categorical variables | *n* | (%) |  |  |  |  |
| Sex |  |  |  |  |  |  |
| Male | 477 | (45.30) |  |  |  |  |
| Female | 577 | (54.70) |  |  |  |  |
| Education level |  |  |  |  |  |  |
| College education and above | 464 | (44.00) |  |  |  |  |
| High school | 238 | (22.60) |  |  |  |  |
| No high school degree | 51 | (4.84) |  |  |  |  |
| Some college | 300 | (28.50) |  |  |  |  |
| Declined to disclose | 1 | (0.09) |  |  |  |  |
| Race |  |  |  |  |  |  |
| Multiracial | 8 | (0.76) |  |  |  |  |
| White | 961 | (91.20) |  |  |  |  |
| African American | 29 | (2.75) |  |  |  |  |
| Native American or Pacific Islander | 5 | (0.47) |  |  |  |  |
| Asian | 2 | (0.19) |  |  |  |  |
| Others | 19 | (1.80) |  |  |  |  |
| Declined to disclose | 30 | (2.85) |  |  |  |  |

*Note*. W2, wave 2 (2004–2006); W1, wave 1 (1995–1996); W3, wave 3 (2013–2014).

Table S2

*W2 Resting State Actigraphy as a Mediator of W1 Child Abuse Predicting W3 Panic Disorder Severity (N = 1,054)*

| Mediator Label | Predictor | a Path (β (SE)) | a Path p | b Path (β (SE)) | b Path p |
| --- | --- | --- | --- | --- | --- |
| Total Activity Counts | Maternal Abuse | 123.791 (SE = 111.313) | .266 | 0 (SE = 0) | **.000** |
| Total Activity Counts | Paternal Abuse | 469.792 (SE = 100.834) | **.000** | 0 (SE = 0) | **.000** |
| Average Activity Counts/min | Maternal Abuse | 0.277 (SE = 0.226) | .221 | 0.017 (SE = 0.002) | **.000** |
| Average Activity Counts/min | Paternal Abuse | 0.914 (SE = 0.204) | **.000** | 0.017 (SE = 0.002) | **.000** |
| Max Activity Counts | Maternal Abuse | 2.235 (SE = 2.332) | .338 | 0.001 (SE = 0) | **.000** |
| Max Activity Counts | Paternal Abuse | 5.25 (SE = 2.112) | **.013** | 0.001 (SE = 0) | **.000** |
| Wake Time | Maternal Abuse | 0.033 (SE = 0.422) | .938 | 0.009 (SE = 0.001) | **.000** |
| Wake Time | Paternal Abuse | 1.93 (SE = 0.382) | **.000** | 0.009 (SE = 0.001) | **.000** |
| % Wake Time | Maternal Abuse | 0.015 (SE = 0.082) | .859 | 0.045 (SE = 0.007) | **.000** |
| % Wake Time | Paternal Abuse | 0.353 (SE = 0.075) | **.000** | 0.045 (SE = 0.007) | **.000** |
| # Wake Bouts | Maternal Abuse | -0.112 (SE = 0.155) | .468 | 0.016 (SE = 0.004) | **.000** |
| # Wake Bouts | Paternal Abuse | 0.562 (SE = 0.14) | **.000** | 0.016 (SE = 0.004) | **.000** |
| Average Sleep Bouts | Maternal Abuse | 0.043 (SE = 0.07) | .535 | -0.013 (SE = 0.008) | .089 |
| Average Sleep Bouts | Paternal Abuse | -0.192 (SE = 0.063) | **.002** | -0.013 (SE = 0.008) | .089 |
| Mediator Label | Predictor | ACME [95% CI] | ACME p | ADE [95% CI] | ADE p |
| Total Activity Counts | Maternal Abuse | 0.004 [-0.004, 0.012] | .304 | 0.014 [-0.021, 0.05] | .444 |
| Total Activity Counts | Paternal Abuse | 0.017 [0.009, 0.026] | **.000** | -0.004 [-0.041, 0.033] | .840 |
| Average Activity Counts/min | Maternal Abuse | 0.005 [-0.003, 0.014] | .292 | 0.017 [-0.023, 0.055] | .400 |
| Average Activity Counts/min | Paternal Abuse | 0.016 [0.007, 0.026] | **.000** | -0.003 [-0.043, 0.034] | .884 |
| Max Activity Counts | Maternal Abuse | 0.002 [-0.004, 0.008] | .428 | 0.019 [-0.017, 0.06] | .332 |
| Max Activity Counts | Paternal Abuse | 0.006 [0.001, 0.011] | **.016** | 0.009 [-0.027, 0.046] | .660 |
| Wake Time | Maternal Abuse | 0 [-0.008, 0.008] | .972 | 0.02 [-0.015, 0.056] | .276 |
| Wake Time | Paternal Abuse | 0.019 [0.01, 0.028] | **.000** | -0.004 [-0.042, 0.033] | .796 |
| % Wake Time | Maternal Abuse | 0.001 [-0.006, 0.009] | .880 | 0.019 [-0.02, 0.062] | .320 |
| % Wake Time | Paternal Abuse | 0.016 [0.008, 0.025] | **.000** | -0.003 [-0.039, 0.034] | .856 |
| # Wake Bouts | Maternal Abuse | -0.002 [-0.008, 0.004] | .628 | 0.021 [-0.023, 0.063] | .308 |
| # Wake Bouts | Paternal Abuse | 0.009 [0.004, 0.015] | **.000** | 0.004 [-0.032, 0.037] | .856 |
| Average Sleep Bouts | Maternal Abuse | -0.001 [-0.003, 0.001] | .496 | 0.02 [-0.021, 0.061] | .344 |
| Average Sleep Bouts | Paternal Abuse | 0.003 [0, 0.006] | .088 | 0.011 [-0.023, 0.046] | .536 |
| Mediator Label | Predictor | Total Effect [95% CI] | Total Effect p | Prop. Mediated [95% CI] | Prop. Mediated p |
| Total Activity Counts | Maternal Abuse | 0.018 [-0.018, 0.054] | .340 | 0.169 [-2.089, 2.256] | .420 |
| Total Activity Counts | Paternal Abuse | 0.012 [-0.022, 0.05] | .552 | 0.611 [-11.441, 12.293] | .552 |
| Average Activity Counts/min | Maternal Abuse | 0.022 [-0.021, 0.061] | .312 | 0.158 [-1.217, 1.914] | .380 |
| Average Activity Counts/min | Paternal Abuse | 0.013 [-0.027, 0.055] | .524 | 0.572 [-9.743, 7.998] | .524 |
| Max Activity Counts | Maternal Abuse | 0.021 [-0.016, 0.065] | .288 | 0.07 [-1.041, 1.094] | .540 |
| Max Activity Counts | Paternal Abuse | 0.014 [-0.021, 0.053] | .420 | 0.214 [-2.12, 6.325] | .436 |
| Wake Time | Maternal Abuse | 0.02 [-0.017, 0.058] | .264 | 0.022 [-1.369, 1.288] | .908 |
| Wake Time | Paternal Abuse | 0.014 [-0.025, 0.052] | .456 | 0.709 [-8.835, 9.701] | .456 |
| % Wake Time | Maternal Abuse | 0.02 [-0.022, 0.063] | .324 | 0.028 [-0.81, 1.543] | .860 |
| % Wake Time | Paternal Abuse | 0.013 [-0.025, 0.051] | .456 | 0.671 [-6.046, 11.301] | .456 |
| # Wake Bouts | Maternal Abuse | 0.019 [-0.025, 0.062] | .328 | -0.044 [-1.277, 1.418] | .804 |
| # Wake Bouts | Paternal Abuse | 0.013 [-0.023, 0.049] | .496 | 0.346 [-6.042, 5.756] | .496 |
| Average Sleep Bouts | Maternal Abuse | 0.019 [-0.021, 0.06] | .340 | -0.012 [-0.442, 0.244] | .716 |
| Average Sleep Bouts | Paternal Abuse | 0.013 [-0.02, 0.049] | .460 | 0.097 [-1.754, 2.253] | .476 |

*Note*. W2, wave 2 (2004–2006); W1, wave 1 (1995–1996); W3, wave 3 (2013–2014); B, standardized beta regression; SE, standard error; %, Percentage of; #, Counts of; ACME, average causal mediation effect; CI, confidence interval; ADE, average direct effect; Prop., Proportion. All analyses adjusted for these W1 covariates: panic disorder severity; generalized anxiety disorder severity; major depressive disorder severity; college education status; total household income; and parental psychopathology status.

Table S3

*W2 Sleeping State Actigraphy as a Mediator of W1 Child Abuse Predicting W3 Panic Disorder Severity (N = 1,054)*

| Mediator Label | Predictor | a Path (β (SE)) | a Path p | b Path (β (SE)) | b Path p |
| --- | --- | --- | --- | --- | --- |
| Total Activity Counts | Maternal Abuse | 43.889 (SE = 62.951) | .486 | 0 (SE = 0) | **.000** |
| Total Activity Counts | Paternal Abuse | 270.601 (SE = 57.025) | **.000** | 0 (SE = 0) | **.000** |
| Average Activity Counts/min | Maternal Abuse | 0.087 (SE = 0.142) | .539 | 0.027 (SE = 0.004) | **.000** |
| Average Activity Counts/min | Paternal Abuse | 0.595 (SE = 0.129) | **.000** | 0.027 (SE = 0.004) | **.000** |
| Max Activity Counts | Maternal Abuse | 1.741 (SE = 2.113) | .410 | 0.001 (SE = 0) | **.000** |
| Max Activity Counts | Paternal Abuse | 4.573 (SE = 1.914) | **.017** | 0.001 (SE = 0) | **.000** |
| Wake Time | Maternal Abuse | 0.058 (SE = 0.294) | .844 | 0.012 (SE = 0.002) | **.000** |
| Wake Time | Paternal Abuse | 1.243 (SE = 0.267) | **.000** | 0.012 (SE = 0.002) | **.000** |
| % Wake Time | Maternal Abuse | 0.01 (SE = 0.065) | .881 | 0.053 (SE = 0.008) | **.000** |
| % Wake Time | Paternal Abuse | 0.263 (SE = 0.059) | **.000** | 0.053 (SE = 0.008) | **.000** |
| # Wake Bouts | Maternal Abuse | -0.088 (SE = 0.133) | .506 | 0.016 (SE = 0.004) | **.000** |
| # Wake Bouts | Paternal Abuse | 0.472 (SE = 0.12) | **.000** | 0.016 (SE = 0.004) | **.000** |
| Average Sleep Bouts | Maternal Abuse | 0.013 (SE = 0.078) | .862 | -0.014 (SE = 0.007) | **.049** |
| Average Sleep Bouts | Paternal Abuse | -0.217 (SE = 0.07) | **.002** | -0.014 (SE = 0.007) | **.049** |
| Mediator Label | Predictor | ACME [95% CI] | ACME p | ADE [95% CI] | ADE p |
| Total Activity Counts | Maternal Abuse | 0.003 [-0.005, 0.011] | .488 | 0.018 [-0.02, 0.054] | .316 |
| Total Activity Counts | Paternal Abuse | 0.017 [0.008, 0.026] | **.000** | -0.003 [-0.04, 0.031] | .876 |
| Average Activity Counts/min | Maternal Abuse | 0.002 [-0.005, 0.009] | .560 | 0.017 [-0.022, 0.054] | .392 |
| Average Activity Counts/min | Paternal Abuse | 0.016 [0.008, 0.027] | **.000** | -0.004 [-0.04, 0.032] | .804 |
| Max Activity Counts | Maternal Abuse | 0.002 [-0.003, 0.008] | .428 | 0.019 [-0.016, 0.054] | .368 |
| Max Activity Counts | Paternal Abuse | 0.005 [0, 0.011] | **.036** | 0.008 [-0.027, 0.045] | .696 |
| Wake Time | Maternal Abuse | 0.001 [-0.006, 0.008] | .880 | 0.019 [-0.021, 0.061] | .296 |
| Wake Time | Paternal Abuse | 0.015 [0.007, 0.025] | **.000** | -0.001 [-0.037, 0.034] | .924 |
| % Wake Time | Maternal Abuse | 0.001 [-0.007, 0.008] | .800 | 0.02 [-0.02, 0.06] | .340 |
| % Wake Time | Paternal Abuse | 0.014 [0.006, 0.023] | **.000** | 0 [-0.035, 0.04] | .948 |
| # Wake Bouts | Maternal Abuse | -0.001 [-0.007, 0.004] | .504 | 0.021 [-0.017, 0.06] | .336 |
| # Wake Bouts | Paternal Abuse | 0.008 [0.003, 0.014] | **.000** | 0.005 [-0.035, 0.042] | .784 |
| Average Sleep Bouts | Maternal Abuse | 0 [-0.002, 0.002] | .864 | 0.02 [-0.018, 0.059] | .316 |
| Average Sleep Bouts | Paternal Abuse | 0.003 [0, 0.007] | **.028** | 0.013 [-0.022, 0.05] | .468 |
| Mediator Label | Predictor | Total Effect [95% CI] | Total Effect p | Prop. Mediated [95% CI] | Prop. Mediated p |
| Total Activity Counts | Maternal Abuse | 0.021 [-0.017, 0.06] | .288 | 0.099 [-1.958, 1.172] | .560 |
| Total Activity Counts | Paternal Abuse | 0.013 [-0.023, 0.05] | .480 | 0.681 [-10.466, 11.32] | .480 |
| Average Activity Counts/min | Maternal Abuse | 0.019 [-0.019, 0.056] | .332 | 0.084 [-1.429, 2.176] | .628 |
| Average Activity Counts/min | Paternal Abuse | 0.012 [-0.025, 0.05] | .524 | 0.641 [-14.342, 10.467] | .524 |
| Max Activity Counts | Maternal Abuse | 0.021 [-0.015, 0.056] | .292 | 0.061 [-0.897, 2.157] | .560 |
| Max Activity Counts | Paternal Abuse | 0.013 [-0.023, 0.049] | .492 | 0.168 [-3.649, 4.648] | .504 |
| Wake Time | Maternal Abuse | 0.02 [-0.022, 0.06] | .292 | 0.028 [-1.314, 1.447] | .868 |
| Wake Time | Paternal Abuse | 0.013 [-0.022, 0.049] | .468 | 0.571 [-6.735, 8.058] | .468 |
| % Wake Time | Maternal Abuse | 0.02 [-0.021, 0.062] | .308 | 0.049 [-1.653, 1.708] | .748 |
| % Wake Time | Paternal Abuse | 0.014 [-0.021, 0.055] | .512 | 0.522 [-8.591, 5.981] | .512 |
| # Wake Bouts | Maternal Abuse | 0.019 [-0.02, 0.06] | .352 | -0.036 [-1.129, 0.947] | .744 |
| # Wake Bouts | Paternal Abuse | 0.012 [-0.028, 0.051] | .552 | 0.274 [-4.971, 2.695] | .552 |
| Average Sleep Bouts | Maternal Abuse | 0.02 [-0.018, 0.059] | .324 | -0.003 [-0.525, 0.352] | .916 |
| Average Sleep Bouts | Paternal Abuse | 0.016 [-0.019, 0.055] | .412 | 0.114 [-1.329, 1.086] | .416 |

*Note*. W2, wave 2 (2004–2006); W1, wave 1 (1995–1996); W3, wave 3 (2013–2014); B, standardized beta regression; SE, standard error; %, Percentage of; #, Counts of; ACME, average causal mediation effect; CI, confidence interval; ADE, average direct effect; Prop., Proportion. All analyses adjusted for these W1 covariates: panic disorder severity; generalized anxiety disorder severity; major depressive disorder severity; college education status; total household income; and parental psychopathology status.

Table S4

*W2 Active Wake State Actigraphy as a Mediator of W1 Child Abuse Predicting W3 Panic Disorder Severity (N = 1,054)*

| Mediator Label | Predictor | a Path (β (SE)) | a Path p | b Path (β (SE)) | b Path p |
| --- | --- | --- | --- | --- | --- |
| Total Activity Counts | Maternal Abuse | 3646.316 (SE = 1106.679) | **.001** | 0 (SE = 0) | .337 |
| Total Activity Counts | Paternal Abuse | 1738.419 (SE = 1002.501) | .083 | 0 (SE = 0) | .337 |
| Average Activity Counts/min | Maternal Abuse | 3.823 (SE = 1.119) | **.001** | 0.001 (SE = 0) | .218 |
| Average Activity Counts/min | Paternal Abuse | 1.901 (SE = 1.014) | .061 | 0.001 (SE = 0) | .218 |
| Max Activity Counts | Maternal Abuse | 3.734 (SE = 3.762) | .321 | 0 (SE = 0) | .225 |
| Max Activity Counts | Paternal Abuse | 12.107 (SE = 3.408) | **.000** | 0 (SE = 0) | .225 |
| Wake Time | Maternal Abuse | 1.439 (SE = 1.097) | .190 | 0 (SE = 0.001) | .510 |
| Wake Time | Paternal Abuse | -0.738 (SE = 0.994) | .458 | 0 (SE = 0.001) | .510 |
| % Wake Time | Maternal Abuse | 0.128 (SE = 0.102) | .211 | 0 (SE = 0.005) | .985 |
| % Wake Time | Paternal Abuse | -0.049 (SE = 0.092) | .599 | 0 (SE = 0.005) | .985 |
| # Wake Bouts | Maternal Abuse | -0.615 (SE = 0.325) | .059 | -0.003 (SE = 0.002) | .131 |
| # Wake Bouts | Paternal Abuse | -0.306 (SE = 0.295) | .299 | -0.003 (SE = 0.002) | .131 |
| Average Sleep Bouts | Maternal Abuse | 0.018 (SE = 0.014) | .202 | 0.187 (SE = 0.038) | **.000** |
| Average Sleep Bouts | Paternal Abuse | 0.011 (SE = 0.013) | .411 | 0.187 (SE = 0.038) | **.000** |
| Mediator Label | Predictor | ACME [95% CI] | ACME p | ADE [95% CI] | ADE p |
| Total Activity Counts | Maternal Abuse | 0.002 [-0.002, 0.007] | .348 | 0.018 [-0.019, 0.056] | .356 |
| Total Activity Counts | Paternal Abuse | 0.001 [-0.001, 0.004] | .480 | 0.012 [-0.023, 0.046] | .556 |
| Average Activity Counts/min | Maternal Abuse | 0.002 [-0.002, 0.008] | .244 | 0.018 [-0.019, 0.057] | .384 |
| Average Activity Counts/min | Paternal Abuse | 0.001 [-0.001, 0.004] | .264 | 0.012 [-0.024, 0.048] | .504 |
| Max Activity Counts | Maternal Abuse | 0.001 [-0.001, 0.003] | .556 | 0.02 [-0.019, 0.061] | .368 |
| Max Activity Counts | Paternal Abuse | 0.002 [-0.001, 0.007] | .276 | 0.011 [-0.028, 0.047] | .532 |
| Wake Time | Maternal Abuse | 0 [-0.003, 0.001] | .564 | 0.021 [-0.013, 0.058] | .288 |
| Wake Time | Paternal Abuse | 0 [-0.001, 0.002] | .652 | 0.013 [-0.021, 0.051] | .484 |
| % Wake Time | Maternal Abuse | 0 [-0.001, 0.001] | .936 | 0.021 [-0.021, 0.061] | .316 |
| % Wake Time | Paternal Abuse | 0 [-0.001, 0.001] | .952 | 0.014 [-0.022, 0.051] | .476 |
| # Wake Bouts | Maternal Abuse | 0.001 [-0.001, 0.005] | .248 | 0.018 [-0.022, 0.059] | .328 |
| # Wake Bouts | Paternal Abuse | 0.001 [-0.001, 0.003] | .384 | 0.012 [-0.029, 0.051] | .520 |
| Average Sleep Bouts | Maternal Abuse | 0.003 [0, 0.008] | .076 | 0.018 [-0.022, 0.054] | .376 |
| Average Sleep Bouts | Paternal Abuse | 0.002 [-0.004, 0.008] | .404 | 0.012 [-0.026, 0.046] | .536 |
| Mediator Label | Predictor | Total Effect [95% CI] | Total Effect p | Prop. Mediated [95% CI] | Prop. Mediated p |
| Total Activity Counts | Maternal Abuse | 0.02 [-0.018, 0.058] | .308 | 0.044 [-0.868, 0.913] | .568 |
| Total Activity Counts | Paternal Abuse | 0.012 [-0.023, 0.047] | .552 | 0.02 [-0.643, 0.65] | .696 |
| Average Activity Counts/min | Maternal Abuse | 0.021 [-0.017, 0.059] | .336 | 0.066 [-1.16, 0.895] | .532 |
| Average Activity Counts/min | Paternal Abuse | 0.013 [-0.023, 0.048] | .460 | 0.032 [-0.723, 1.005] | .580 |
| Max Activity Counts | Maternal Abuse | 0.02 [-0.018, 0.062] | .344 | 0.012 [-0.265, 0.475] | .716 |
| Max Activity Counts | Paternal Abuse | 0.013 [-0.026, 0.05] | .468 | 0.069 [-1.196, 1.481] | .592 |
| Wake Time | Maternal Abuse | 0.021 [-0.014, 0.057] | .296 | -0.01 [-0.424, 0.368] | .684 |
| Wake Time | Paternal Abuse | 0.014 [-0.02, 0.051] | .476 | 0.003 [-0.292, 0.319] | .808 |
| % Wake Time | Maternal Abuse | 0.021 [-0.021, 0.062] | .312 | 0 [-0.191, 0.209] | .944 |
| % Wake Time | Paternal Abuse | 0.014 [-0.022, 0.051] | .472 | 0 [-0.145, 0.139] | .976 |
| # Wake Bouts | Maternal Abuse | 0.02 [-0.021, 0.061] | .312 | 0.038 [-0.581, 0.469] | .440 |
| # Wake Bouts | Paternal Abuse | 0.013 [-0.028, 0.051] | .520 | 0.018 [-0.486, 0.43] | .688 |
| Average Sleep Bouts | Maternal Abuse | 0.021 [-0.018, 0.059] | .296 | 0.099 [-0.979, 1.895] | .364 |
| Average Sleep Bouts | Paternal Abuse | 0.014 [-0.022, 0.049] | .444 | 0.072 [-1.567, 1.814] | .664 |

*Note*. W2, wave 2 (2004–2006); W1, wave 1 (1995–1996); W3, wave 3 (2013–2014); B, standardized beta regression; SE, standard error; %, Percentage of; #, Counts of; ACME, average causal mediation effect; CI, confidence interval; ADE, average direct effect; Prop., Proportion. All analyses adjusted for these W1 covariates: panic disorder severity; generalized anxiety disorder severity; major depressive disorder severity; college education status; total household income; and parental psychopathology status.

Table S5

*W2 Resting State Actigraphy as a Mediator of W1 Child Affection Predicting W3 Panic Disorder Severity (N = 1,054)*

| Mediator Label | Predictor | a Path (β (SE)) | a Path p | b Path (β (SE)) | b Path p |
| --- | --- | --- | --- | --- | --- |
| Total Activity Counts | Maternal Affection | -80.622 (SE = 40.639) | **.048** | 0 (SE = 0) | **.000** |
| Total Activity Counts | Paternal Affection | 40.404 (SE = 22.388) | .071 | 0 (SE = 0) | **.000** |
| Average Activity Counts/min | Maternal Affection | -0.163 (SE = 0.082) | **.048** | 0.017 (SE = 0.002) | **.000** |
| Average Activity Counts/min | Paternal Affection | 0.084 (SE = 0.045) | .066 | 0.017 (SE = 0.002) | **.000** |
| Max Activity Counts | Maternal Affection | -1.263 (SE = 0.844) | .135 | 0.001 (SE = 0) | **.000** |
| Max Activity Counts | Paternal Affection | 0.621 (SE = 0.465) | .182 | 0.001 (SE = 0) | **.000** |
| Wake Time | Maternal Affection | -0.219 (SE = 0.154) | .155 | 0.009 (SE = 0.001) | **.000** |
| Wake Time | Paternal Affection | 0.189 (SE = 0.085) | **.026** | 0.009 (SE = 0.001) | **.000** |
| % Wake Time | Maternal Affection | -0.044 (SE = 0.03) | .141 | 0.045 (SE = 0.006) | **.000** |
| % Wake Time | Paternal Affection | 0.038 (SE = 0.017) | **.021** | 0.045 (SE = 0.006) | **.000** |
| # Wake Bouts | Maternal Affection | -0.032 (SE = 0.056) | .568 | 0.015 (SE = 0.004) | **.000** |
| # Wake Bouts | Paternal Affection | 0.095 (SE = 0.031) | **.002** | 0.015 (SE = 0.004) | **.000** |
| Average Sleep Bouts | Maternal Affection | 0.028 (SE = 0.025) | .263 | -0.013 (SE = 0.008) | .090 |
| Average Sleep Bouts | Paternal Affection | -0.016 (SE = 0.014) | .251 | -0.013 (SE = 0.008) | .090 |
| Mediator Label | Predictor | ACME [95% CI] | ACME p | ADE [95% CI] | ADE p |
| Total Activity Counts | Maternal Affection | -0.003 [-0.006, 0] | .060 | -0.008 [-0.019, 0.004] | .216 |
| Total Activity Counts | Paternal Affection | 0.001 [0, 0.003] | **.040** | 0.006 [-0.002, 0.014] | .180 |
| Average Activity Counts/min | Maternal Affection | -0.003 [-0.006, 0] | .060 | -0.008 [-0.021, 0.003] | .224 |
| Average Activity Counts/min | Paternal Affection | 0.001 [0, 0.003] | **.040** | 0.006 [-0.002, 0.014] | .172 |
| Max Activity Counts | Maternal Affection | -0.001 [-0.004, 0.001] | .204 | -0.009 [-0.02, 0.002] | .148 |
| Max Activity Counts | Paternal Affection | 0.001 [0, 0.002] | .180 | 0.007 [-0.002, 0.014] | .108 |
| Wake Time | Maternal Affection | -0.002 [-0.005, 0.001] | .176 | -0.008 [-0.02, 0.003] | .188 |
| Wake Time | Paternal Affection | 0.002 [0, 0.003] | **.012** | 0.006 [-0.002, 0.013] | .152 |
| % Wake Time | Maternal Affection | -0.002 [-0.006, 0.001] | .184 | -0.008 [-0.021, 0.004] | .160 |
| % Wake Time | Paternal Affection | 0.002 [0, 0.003] | **.024** | 0.005 [-0.003, 0.013] | .192 |
| # Wake Bouts | Maternal Affection | 0 [-0.002, 0.001] | .564 | -0.01 [-0.022, 0.003] | .132 |
| # Wake Bouts | Paternal Affection | 0.001 [0, 0.003] | **.008** | 0.006 [-0.003, 0.014] | .176 |
| Average Sleep Bouts | Maternal Affection | 0 [-0.001, 0] | .192 | -0.01 [-0.022, 0.001] | .096 |
| Average Sleep Bouts | Paternal Affection | 0 [0, 0.001] | .204 | 0.007 [-0.001, 0.016] | .068 |
| Mediator Label | Predictor | Total Effect [95% CI] | Total Effect p | Prop. Mediated [95% CI] | Prop. Mediated p |
| Total Activity Counts | Maternal Affection | -0.01 [-0.022, 0.002] | .108 | 0.231 [-0.758, 1.285] | .160 |
| Total Activity Counts | Paternal Affection | 0.007 [-0.001, 0.015] | .080 | 0.19 [-0.748, 1.409] | .112 |
| Average Activity Counts/min | Maternal Affection | -0.011 [-0.024, 0.002] | .104 | 0.251 [-0.924, 1.424] | .140 |
| Average Activity Counts/min | Paternal Affection | 0.007 [-0.001, 0.015] | .084 | 0.183 [-0.419, 1.644] | .108 |
| Max Activity Counts | Maternal Affection | -0.01 [-0.022, 0.001] | .084 | 0.119 [-0.515, 1.493] | .264 |
| Max Activity Counts | Paternal Affection | 0.007 [-0.001, 0.015] | .072 | 0.087 [-0.091, 0.757] | .220 |
| Wake Time | Maternal Affection | -0.011 [-0.022, 0.002] | .108 | 0.183 [-0.703, 1.338] | .244 |
| Wake Time | Paternal Affection | 0.007 [0, 0.016] | .060 | 0.23 [-0.579, 1.296] | .072 |
| % Wake Time | Maternal Affection | -0.01 [-0.022, 0.003] | .088 | 0.187 [-0.374, 1.487] | .216 |
| % Wake Time | Paternal Affection | 0.007 [-0.001, 0.015] | .072 | 0.221 [-0.805, 2.246] | .088 |
| # Wake Bouts | Maternal Affection | -0.011 [-0.022, 0.002] | .112 | 0.03 [-0.361, 0.495] | .604 |
| # Wake Bouts | Paternal Affection | 0.007 [-0.001, 0.015] | .124 | 0.181 [-1.558, 0.985] | .132 |
| Average Sleep Bouts | Maternal Affection | -0.011 [-0.022, 0.001] | .080 | 0.028 [-0.05, 0.287] | .272 |
| Average Sleep Bouts | Paternal Affection | 0.007 [-0.001, 0.016] | .060 | 0.025 [-0.055, 0.279] | .248 |

*Note*. W2, wave 2 (2004–2006); W1, wave 1 (1995–1996); W3, wave 3 (2013–2014); B, standardized beta regression; SE, standard error; %, Percentage of; #, Counts of; ACME, average causal mediation effect; CI, confidence interval; ADE, average direct effect; Prop., Proportion. All analyses adjusted for these W1 covariates: panic disorder severity; generalized anxiety disorder severity; major depressive disorder severity; college education status; total household income; and parental psychopathology status.

Table S6

*W2 Sleeping State Actigraphy as a Mediator of W1 Child Affection Predicting W3 Panic Disorder Severity (N = 1,054)*

| Mediator Label | Predictor | a Path (β (SE)) | a Path p | b Path (β (SE)) | b Path p |
| --- | --- | --- | --- | --- | --- |
| Total Activity Counts | Maternal Affection | -42.928 (SE = 22.935) | .062 | 0 (SE = 0) | **.000** |
| Total Activity Counts | Paternal Affection | 32.238 (SE = 12.635) | **.011** | 0 (SE = 0) | **.000** |
| Average Activity Counts/min | Maternal Affection | -0.094 (SE = 0.052) | .070 | 0.026 (SE = 0.004) | **.000** |
| Average Activity Counts/min | Paternal Affection | 0.077 (SE = 0.028) | **.007** | 0.026 (SE = 0.004) | **.000** |
| Max Activity Counts | Maternal Affection | -1.237 (SE = 0.764) | .105 | 0.001 (SE = 0) | **.000** |
| Max Activity Counts | Paternal Affection | 0.732 (SE = 0.421) | .082 | 0.001 (SE = 0) | **.000** |
| Wake Time | Maternal Affection | -0.166 (SE = 0.107) | .121 | 0.012 (SE = 0.002) | **.000** |
| Wake Time | Paternal Affection | 0.164 (SE = 0.059) | **.005** | 0.012 (SE = 0.002) | **.000** |
| % Wake Time | Maternal Affection | -0.035 (SE = 0.024) | .133 | 0.053 (SE = 0.008) | **.000** |
| % Wake Time | Paternal Affection | 0.038 (SE = 0.013) | **.003** | 0.053 (SE = 0.008) | **.000** |
| # Wake Bouts | Maternal Affection | -0.035 (SE = 0.048) | .472 | 0.016 (SE = 0.004) | **.000** |
| # Wake Bouts | Paternal Affection | 0.061 (SE = 0.026) | **.021** | 0.016 (SE = 0.004) | **.000** |
| Average Sleep Bouts | Maternal Affection | 0.046 (SE = 0.028) | .100 | -0.014 (SE = 0.007) | .051 |
| Average Sleep Bouts | Paternal Affection | -0.021 (SE = 0.015) | .170 | -0.014 (SE = 0.007) | .051 |
| Mediator Label | Predictor | ACME [95% CI] | ACME p | ADE [95% CI] | ADE p |
| Total Activity Counts | Maternal Affection | -0.003 [-0.006, 0.001] | .160 | -0.008 [-0.019, 0.004] | .196 |
| Total Activity Counts | Paternal Affection | 0.002 [0.001, 0.004] | **.008** | 0.005 [-0.002, 0.013] | .208 |
| Average Activity Counts/min | Maternal Affection | -0.003 [-0.006, 0.001] | .116 | -0.008 [-0.02, 0.005] | .204 |
| Average Activity Counts/min | Paternal Affection | 0.002 [0.001, 0.004] | **.004** | 0.005 [-0.002, 0.013] | .264 |
| Max Activity Counts | Maternal Affection | -0.001 [-0.004, 0] | .172 | -0.009 [-0.022, 0.002] | .120 |
| Max Activity Counts | Paternal Affection | 0.001 [0, 0.002] | .084 | 0.007 [-0.001, 0.015] | .108 |
| Wake Time | Maternal Affection | -0.002 [-0.006, 0.001] | .188 | -0.009 [-0.02, 0.003] | .168 |
| Wake Time | Paternal Affection | 0.002 [0, 0.003] | **.008** | 0.005 [-0.003, 0.013] | .256 |
| % Wake Time | Maternal Affection | -0.002 [-0.005, 0.001] | .192 | -0.009 [-0.02, 0.003] | .144 |
| % Wake Time | Paternal Affection | 0.002 [0.001, 0.004] | **.004** | 0.005 [-0.002, 0.012] | .204 |
| # Wake Bouts | Maternal Affection | -0.001 [-0.002, 0.001] | .496 | -0.01 [-0.022, 0.002] | .104 |
| # Wake Bouts | Paternal Affection | 0.001 [0, 0.002] | **.016** | 0.006 [-0.002, 0.013] | .148 |
| Average Sleep Bouts | Maternal Affection | -0.001 [-0.002, 0] | .100 | -0.01 [-0.022, 0.003] | .112 |
| Average Sleep Bouts | Paternal Affection | 0 [0, 0.001] | .156 | 0.006 [-0.001, 0.014] | .096 |
| Mediator Label | Predictor | Total Effect [95% CI] | Total Effect p | Prop. Mediated [95% CI] | Prop. Mediated p |
| Total Activity Counts | Maternal Affection | -0.01 [-0.023, 0.003] | .108 | 0.226 [-0.805, 1.343] | .228 |
| Total Activity Counts | Paternal Affection | 0.007 [-0.001, 0.015] | .076 | 0.278 [-0.69, 1.62] | .084 |
| Average Activity Counts/min | Maternal Affection | -0.01 [-0.022, 0.002] | .104 | 0.22 [-0.945, 1.506] | .188 |
| Average Activity Counts/min | Paternal Affection | 0.007 [-0.001, 0.015] | .096 | 0.282 [-1.231, 2.941] | .100 |
| Max Activity Counts | Maternal Affection | -0.011 [-0.023, 0.001] | .076 | 0.116 [-0.239, 0.938] | .224 |
| Max Activity Counts | Paternal Affection | 0.007 [-0.001, 0.016] | .084 | 0.098 [-0.304, 0.7] | .160 |
| Wake Time | Maternal Affection | -0.011 [-0.023, 0.001] | .096 | 0.167 [-0.752, 1.345] | .244 |
| Wake Time | Paternal Affection | 0.007 [-0.001, 0.014] | .120 | 0.248 [-1.161, 1.513] | .112 |
| % Wake Time | Maternal Affection | -0.011 [-0.022, 0.001] | .080 | 0.158 [-0.362, 1.159] | .240 |
| % Wake Time | Paternal Affection | 0.007 [0, 0.015] | .052 | 0.254 [-0.433, 2.348] | .056 |
| # Wake Bouts | Maternal Affection | -0.011 [-0.022, 0.001] | .088 | 0.045 [-0.407, 0.618] | .496 |
| # Wake Bouts | Paternal Affection | 0.007 [-0.001, 0.014] | .084 | 0.129 [-0.411, 1.063] | .092 |
| Average Sleep Bouts | Maternal Affection | -0.01 [-0.023, 0.002] | .096 | 0.046 [-0.208, 0.424] | .172 |
| Average Sleep Bouts | Paternal Affection | 0.007 [-0.001, 0.014] | .068 | 0.039 [-0.06, 0.313] | .216 |

*Note*. W2, wave 2 (2004–2006); W1, wave 1 (1995–1996); W3, wave 3 (2013–2014); B, standardized beta regression; SE, standard error; %, Percentage of; #, Counts of; ACME, average causal mediation effect; CI, confidence interval; ADE, average direct effect; Prop., Proportion. All analyses adjusted for these W1 covariates: panic disorder severity; generalized anxiety disorder severity; major depressive disorder severity; college education status; total household income; and parental psychopathology status.

Table S7

*W2 Active Wake State Actigraphy as a Mediator of W1 Child Affection Predicting W3 Panic Disorder Severity (N = 1,054)*

| Mediator Label | Predictor | a Path (β (SE)) | a Path p | b Path (β (SE)) | b Path p |
| --- | --- | --- | --- | --- | --- |
| Total Activity Counts | Maternal Affection | -1260.287 (SE = 401.428) | **.002** | 0 (SE = 0) | .288 |
| Total Activity Counts | Paternal Affection | 71.413 (SE = 221.15) | .747 | 0 (SE = 0) | .288 |
| Average Activity Counts/min | Maternal Affection | -1.173 (SE = 0.407) | **.004** | 0.001 (SE = 0) | .173 |
| Average Activity Counts/min | Paternal Affection | 0.033 (SE = 0.224) | .883 | 0.001 (SE = 0) | .173 |
| Max Activity Counts | Maternal Affection | -1.342 (SE = 1.369) | .327 | 0 (SE = 0) | .158 |
| Max Activity Counts | Paternal Affection | -0.353 (SE = 0.754) | .640 | 0 (SE = 0) | .158 |
| Wake Time | Maternal Affection | -0.608 (SE = 0.395) | .125 | 0 (SE = 0.001) | .494 |
| Wake Time | Paternal Affection | -0.027 (SE = 0.218) | .903 | 0 (SE = 0.001) | .494 |
| % Wake Time | Maternal Affection | -0.038 (SE = 0.037) | .304 | 0 (SE = 0.005) | .985 |
| % Wake Time | Paternal Affection | -0.014 (SE = 0.02) | .485 | 0 (SE = 0.005) | .985 |
| # Wake Bouts | Maternal Affection | 0.232 (SE = 0.117) | **.049** | -0.003 (SE = 0.002) | .120 |
| # Wake Bouts | Paternal Affection | -0.027 (SE = 0.065) | .674 | -0.003 (SE = 0.002) | .120 |
| Average Sleep Bouts | Maternal Affection | -0.008 (SE = 0.005) | .108 | 0.185 (SE = 0.038) | **.000** |
| Average Sleep Bouts | Paternal Affection | 0.005 (SE = 0.003) | .059 | 0.185 (SE = 0.038) | **.000** |
| Mediator Label | Predictor | ACME [95% CI] | ACME p | ADE [95% CI] | ADE p |
| Total Activity Counts | Maternal Affection | -0.001 [-0.002, 0.001] | .264 | -0.01 [-0.022, 0.002] | .100 |
| Total Activity Counts | Paternal Affection | 0 [0, 0.001] | .768 | 0.007 [-0.001, 0.015] | .108 |
| Average Activity Counts/min | Maternal Affection | -0.001 [-0.002, 0] | .216 | -0.009 [-0.022, 0.003] | .140 |
| Average Activity Counts/min | Paternal Affection | 0 [0, 0] | .876 | 0.007 [-0.001, 0.015] | .080 |
| Max Activity Counts | Maternal Affection | 0 [-0.001, 0] | .428 | -0.01 [-0.022, 0.002] | .084 |
| Max Activity Counts | Paternal Affection | 0 [-0.001, 0] | .696 | 0.007 [-0.001, 0.015] | .096 |
| Wake Time | Maternal Affection | 0 [0, 0.001] | .444 | -0.011 [-0.023, 0.001] | .084 |
| Wake Time | Paternal Affection | 0 [0, 0] | .936 | 0.007 [-0.001, 0.015] | .080 |
| % Wake Time | Maternal Affection | 0 [-0.001, 0] | .956 | -0.011 [-0.023, 0.001] | .068 |
| % Wake Time | Paternal Affection | 0 [0, 0] | .972 | 0.007 [-0.001, 0.015] | .104 |
| # Wake Bouts | Maternal Affection | -0.001 [-0.002, 0] | .108 | -0.01 [-0.021, 0.001] | .072 |
| # Wake Bouts | Paternal Affection | 0 [0, 0.001] | .708 | 0.007 [-0.001, 0.015] | .064 |
| Average Sleep Bouts | Maternal Affection | -0.002 [-0.004, 0] | .112 | -0.009 [-0.021, 0.004] | .116 |
| Average Sleep Bouts | Paternal Affection | 0.001 [0, 0.002] | **.004** | 0.006 [-0.002, 0.014] | .108 |
| Mediator Label | Predictor | Total Effect [95% CI] | Total Effect p | Prop. Mediated [95% CI] | Prop. Mediated p |
| Total Activity Counts | Maternal Affection | -0.011 [-0.022, 0.001] | .088 | 0.053 [-0.109, 0.6] | .288 |
| Total Activity Counts | Paternal Affection | 0.007 [-0.001, 0.016] | .104 | 0.002 [-0.146, 0.161] | .808 |
| Average Activity Counts/min | Maternal Affection | -0.01 [-0.023, 0.002] | .108 | 0.068 [-0.272, 0.656] | .292 |
| Average Activity Counts/min | Paternal Affection | 0.007 [-0.001, 0.015] | .076 | 0.001 [-0.088, 0.106] | .896 |
| Max Activity Counts | Maternal Affection | -0.01 [-0.022, 0.002] | .076 | 0.019 [-0.066, 0.253] | .464 |
| Max Activity Counts | Paternal Affection | 0.007 [-0.002, 0.015] | .096 | -0.004 [-0.155, 0.102] | .728 |
| Wake Time | Maternal Affection | -0.011 [-0.023, 0.001] | .092 | -0.01 [-0.209, 0.085] | .496 |
| Wake Time | Paternal Affection | 0.007 [-0.001, 0.015] | .080 | 0 [-0.062, 0.066] | .944 |
| % Wake Time | Maternal Affection | -0.011 [-0.023, 0.002] | .068 | 0 [-0.064, 0.082] | .952 |
| % Wake Time | Paternal Affection | 0.007 [-0.001, 0.015] | .104 | 0 [-0.051, 0.073] | .972 |
| # Wake Bouts | Maternal Affection | -0.01 [-0.022, 0.001] | .068 | 0.048 [-0.116, 0.338] | .168 |
| # Wake Bouts | Paternal Affection | 0.007 [-0.001, 0.015] | .060 | 0.008 [-0.082, 0.154] | .736 |
| Average Sleep Bouts | Maternal Affection | -0.011 [-0.024, 0.003] | .092 | 0.125 [-0.444, 0.643] | .204 |
| Average Sleep Bouts | Paternal Affection | 0.007 [-0.001, 0.015] | .076 | 0.132 [-0.576, 0.749] | .080 |

*Note*. W2, wave 2 (2004–2006); W1, wave 1 (1995–1996); W3, wave 3 (2013–2014); B, standardized beta regression; SE, standard error; %, Percentage of; #, Counts of; ACME, average causal mediation effect; CI, confidence interval; ADE, average direct effect; Prop., Proportion. All analyses adjusted for these W1 covariates: panic disorder severity; generalized anxiety disorder severity; major depressive disorder severity; college education status; total household income; and parental psychopathology status.

Table S8

*W2 Resting State Actigraphy as a Mediator of W1 Child Abuse Predicting W3 Panic Disorder Severity (N = 308) (Sensitivity Analysis)*

| Mediator Label | Predictor | a Path (β (SE)) | a Path p | b Path (β (SE)) | b Path p |
| --- | --- | --- | --- | --- | --- |
| Total Activity Counts | Maternal Abuse | -55.884 (SE = 265.705) | .834 | 0 (SE = 0) | **.028** |
| Total Activity Counts | Paternal Abuse | 495.495 (SE = 259.825) | .057 | 0 (SE = 0) | **.028** |
| Average Activity Counts/min | Maternal Abuse | -0.016 (SE = 0.553) | .977 | 0.006 (SE = 0.003) | .061 |
| Average Activity Counts/min | Paternal Abuse | 0.968 (SE = 0.541) | .075 | 0.006 (SE = 0.003) | .061 |
| Max Activity Counts | Maternal Abuse | -1.169 (SE = 6.189) | .850 | 0 (SE = 0) | .328 |
| Max Activity Counts | Paternal Abuse | -0.763 (SE = 6.052) | .900 | 0 (SE = 0) | .328 |
| Wake Time | Maternal Abuse | -1.165 (SE = 0.978) | .235 | 0.004 (SE = 0.002) | **.032** |
| Wake Time | Paternal Abuse | 2.316 (SE = 0.956) | **.016** | 0.004 (SE = 0.002) | **.032** |
| % Wake Time | Maternal Abuse | -0.182 (SE = 0.201) | .367 | 0.016 (SE = 0.009) | .084 |
| % Wake Time | Paternal Abuse | 0.441 (SE = 0.196) | .026 | 0.016 (SE = 0.009) | .084 |
| # Wake Bouts | Maternal Abuse | -0.518 (SE = 0.405) | .201 | 0.007 (SE = 0.005) | .152 |
| # Wake Bouts | Paternal Abuse | 0.757 (SE = 0.396) | .057 | 0.007 (SE = 0.005) | .152 |
| Average Sleep Bouts | Maternal Abuse | 0.148 (SE = 0.199) | .460 | -0.007 (SE = 0.009) | .462 |
| Average Sleep Bouts | Paternal Abuse | -0.387 (SE = 0.195) | **.048** | -0.007 (SE = 0.009) | .462 |
| Mediator Label | Predictor | ACME [95% CI] | ACME p | ADE [95% CI] | ADE p |
| Total Activity Counts | Maternal Abuse | -0.001 [-0.01, 0.009] | .820 | -0.008 [-0.067, 0.052] | .784 |
| Total Activity Counts | Paternal Abuse | 0.008 [-0.001, 0.022] | .112 | -0.026 [-0.095, 0.042] | .492 |
| Average Activity Counts/min | Maternal Abuse | 0 [-0.008, 0.009] | .968 | -0.008 [-0.068, 0.054] | .788 |
| Average Activity Counts/min | Paternal Abuse | 0.006 [-0.001, 0.018] | .120 | -0.03 [-0.107, 0.039] | .436 |
| Max Activity Counts | Maternal Abuse | 0 [-0.008, 0.006] | .948 | -0.006 [-0.069, 0.058] | .868 |
| Max Activity Counts | Paternal Abuse | 0 [-0.006, 0.006] | .856 | -0.024 [-0.1, 0.046] | .488 |
| Wake Time | Maternal Abuse | -0.005 [-0.014, 0.002] | .232 | -0.002 [-0.058, 0.052] | .984 |
| Wake Time | Paternal Abuse | 0.009 [0, 0.023] | **.040** | -0.031 [-0.101, 0.031] | .372 |
| % Wake Time | Maternal Abuse | -0.003 [-0.013, 0.003] | .368 | -0.005 [-0.06, 0.057] | .864 |
| % Wake Time | Paternal Abuse | 0.007 [0, 0.019] | .084 | -0.028 [-0.101, 0.039] | .452 |
| # Wake Bouts | Maternal Abuse | -0.004 [-0.017, 0.003] | .456 | -0.003 [-0.063, 0.057] | .920 |
| # Wake Bouts | Paternal Abuse | 0.005 [-0.003, 0.016] | .228 | -0.026 [-0.099, 0.042] | .476 |
| Average Sleep Bouts | Maternal Abuse | -0.001 [-0.005, 0.002] | .568 | -0.004 [-0.062, 0.056] | .848 |
| Average Sleep Bouts | Paternal Abuse | 0.003 [-0.002, 0.009] | .300 | -0.025 [-0.09, 0.045] | .412 |
| Mediator Label | Predictor | Total Effect [95% CI] | Total Effect p | Prop. Mediated [95% CI] | Prop. Mediated p |
| Total Activity Counts | Maternal Abuse | -0.009 [-0.069, 0.053] | .768 | 0.006 [-1.423, 2.215] | .932 |
| Total Activity Counts | Paternal Abuse | -0.018 [-0.087, 0.05] | .660 | -0.083 [-1.87, 4.8] | .732 |
| Average Activity Counts/min | Maternal Abuse | -0.008 [-0.067, 0.055] | .784 | 0.005 [-1.253, 1.267] | .944 |
| Average Activity Counts/min | Paternal Abuse | -0.024 [-0.101, 0.049] | .512 | -0.077 [-2.26, 1.244] | .584 |
| Max Activity Counts | Maternal Abuse | -0.007 [-0.072, 0.058] | .844 | 0.009 [-0.815, 1.143] | .840 |
| Max Activity Counts | Paternal Abuse | -0.024 [-0.1, 0.046] | .480 | 0.004 [-0.776, 0.486] | .864 |
| Wake Time | Maternal Abuse | -0.006 [-0.066, 0.046] | .916 | 0.026 [-2.795, 1.625] | .900 |
| Wake Time | Paternal Abuse | -0.022 [-0.093, 0.043] | .560 | -0.119 [-2.838, 4.344] | .592 |
| % Wake Time | Maternal Abuse | -0.008 [-0.063, 0.056] | .812 | 0.021 [-2.648, 1.208] | .876 |
| % Wake Time | Paternal Abuse | -0.021 [-0.093, 0.047] | .580 | -0.083 [-2.104, 3.244] | .624 |
| # Wake Bouts | Maternal Abuse | -0.007 [-0.068, 0.055] | .832 | 0.022 [-2.106, 2.405] | .856 |
| # Wake Bouts | Paternal Abuse | -0.021 [-0.095, 0.045] | .572 | -0.05 [-1.675, 1.681] | .712 |
| Average Sleep Bouts | Maternal Abuse | -0.005 [-0.064, 0.057] | .840 | 0.009 [-0.652, 0.469] | .824 |
| Average Sleep Bouts | Paternal Abuse | -0.023 [-0.089, 0.048] | .464 | -0.029 [-0.985, 1.073] | .692 |

*Note*. W2, wave 2 (2004–2006); W1, wave 1 (1995–1996); W3, wave 3 (2013–2014); B, standardized beta regression; SE, standard error; %, Percentage of; #, Counts of; ACME, average causal mediation effect; CI, confidence interval; ADE, average direct effect; Prop., Proportion. All analyses adjusted for these W1 covariates: panic disorder severity; generalized anxiety disorder severity; major depressive disorder severity; college education status; total household income; and parental psychopathology status.

Table S9

*W2 Sleeping State Actigraphy as a Mediator of W1 Child Abuse Predicting W3 Panic Disorder Severity (N = 308) (Sensitivity Analysis)*

| Mediator Label | Predictor | a Path (β (SE)) | a Path p | b Path (β (SE)) | b Path p |
| --- | --- | --- | --- | --- | --- |
| Total Activity Counts | Maternal Abuse | -29.605 (SE = 133.56) | .825 | 0 (SE = 0) | **.045** |
| Total Activity Counts | Paternal Abuse | 276.255 (SE = 130.604) | **.035** | 0 (SE = 0) | **.045** |
| Average Activity Counts/min | Maternal Abuse | -0.112 (SE = 0.313) | .720 | 0.01 (SE = 0.006) | .091 |
| Average Activity Counts/min | Paternal Abuse | 0.608 (SE = 0.306) | .048 | 0.01 (SE = 0.006) | .091 |
| Max Activity Counts | Maternal Abuse | 0.264 (SE = 5.237) | .960 | 0 (SE = 0) | .444 |
| Max Activity Counts | Paternal Abuse | -0.85 (SE = 5.121) | .868 | 0 (SE = 0) | .444 |
| Wake Time | Maternal Abuse | -0.461 (SE = 0.618) | .456 | 0.005 (SE = 0.003) | .072 |
| Wake Time | Paternal Abuse | 1.245 (SE = 0.604) | **.040** | 0.005 (SE = 0.003) | .072 |
| % Wake Time | Maternal Abuse | -0.111 (SE = 0.147) | .449 | 0.019 (SE = 0.013) | .140 |
| % Wake Time | Paternal Abuse | 0.28 (SE = 0.144) | .052 | 0.019 (SE = 0.013) | .140 |
| # Wake Bouts | Maternal Abuse | -0.371 (SE = 0.325) | .254 | 0.008 (SE = 0.006) | .157 |
| # Wake Bouts | Paternal Abuse | 0.584 (SE = 0.317) | .067 | 0.008 (SE = 0.006) | .157 |
| Average Sleep Bouts | Maternal Abuse | 0.07 (SE = 0.216) | .744 | -0.01 (SE = 0.009) | .246 |
| Average Sleep Bouts | Paternal Abuse | -0.418 (SE = 0.211) | **.049** | -0.01 (SE = 0.009) | .246 |
| Mediator Label | Predictor | ACME [95% CI] | ACME p | ADE [95% CI] | ADE p |
| Total Activity Counts | Maternal Abuse | -0.001 [-0.01, 0.006] | .832 | -0.005 [-0.062, 0.055] | .888 |
| Total Activity Counts | Paternal Abuse | 0.008 [-0.002, 0.023] | .164 | -0.031 [-0.105, 0.033] | .348 |
| Average Activity Counts/min | Maternal Abuse | -0.001 [-0.008, 0.005] | .748 | -0.006 [-0.065, 0.063] | .836 |
| Average Activity Counts/min | Paternal Abuse | 0.006 [-0.002, 0.02] | .204 | -0.029 [-0.095, 0.038] | .420 |
| Max Activity Counts | Maternal Abuse | 0 [-0.004, 0.004] | .940 | -0.007 [-0.067, 0.057] | .780 |
| Max Activity Counts | Paternal Abuse | 0 [-0.006, 0.005] | .956 | -0.025 [-0.088, 0.041] | .464 |
| Wake Time | Maternal Abuse | -0.003 [-0.011, 0.003] | .356 | -0.004 [-0.065, 0.059] | .884 |
| Wake Time | Paternal Abuse | 0.007 [-0.001, 0.023] | .136 | -0.029 [-0.101, 0.039] | .408 |
| % Wake Time | Maternal Abuse | -0.002 [-0.009, 0.004] | .452 | -0.002 [-0.06, 0.054] | .936 |
| % Wake Time | Paternal Abuse | 0.005 [-0.003, 0.015] | .224 | -0.029 [-0.094, 0.046] | .444 |
| # Wake Bouts | Maternal Abuse | -0.003 [-0.013, 0.002] | .368 | -0.005 [-0.065, 0.053] | .848 |
| # Wake Bouts | Paternal Abuse | 0.005 [-0.003, 0.017] | .252 | -0.027 [-0.097, 0.046] | .440 |
| Average Sleep Bouts | Maternal Abuse | -0.001 [-0.005, 0.003] | .656 | -0.006 [-0.07, 0.061] | .860 |
| Average Sleep Bouts | Paternal Abuse | 0.004 [-0.002, 0.012] | .188 | -0.027 [-0.094, 0.037] | .428 |
| Mediator Label | Predictor | Total Effect [95% CI] | Total Effect p | Prop. Mediated [95% CI] | Prop. Mediated p |
| Total Activity Counts | Maternal Abuse | -0.006 [-0.065, 0.055] | .888 | 0.011 [-0.946, 0.789] | .864 |
| Total Activity Counts | Paternal Abuse | -0.023 [-0.097, 0.04] | .484 | -0.106 [-4.179, 2.969] | .600 |
| Average Activity Counts/min | Maternal Abuse | -0.007 [-0.067, 0.061] | .792 | 0.008 [-1.262, 0.847] | .916 |
| Average Activity Counts/min | Paternal Abuse | -0.023 [-0.091, 0.046] | .524 | -0.068 [-2.916, 2.646] | .656 |
| Max Activity Counts | Maternal Abuse | -0.007 [-0.067, 0.056] | .772 | 0 [-0.652, 0.518] | .984 |
| Max Activity Counts | Paternal Abuse | -0.025 [-0.088, 0.04] | .448 | 0 [-0.545, 0.426] | .996 |
| Wake Time | Maternal Abuse | -0.007 [-0.07, 0.056] | .824 | 0.018 [-1.142, 2.082] | .884 |
| Wake Time | Paternal Abuse | -0.022 [-0.097, 0.05] | .572 | -0.062 [-1.888, 3.115] | .660 |
| % Wake Time | Maternal Abuse | -0.004 [-0.062, 0.052] | .876 | 0.014 [-1.947, 1.555] | .872 |
| % Wake Time | Paternal Abuse | -0.024 [-0.089, 0.05] | .524 | -0.052 [-2.448, 1.621] | .652 |
| # Wake Bouts | Maternal Abuse | -0.008 [-0.069, 0.052] | .752 | 0.037 [-1.049, 1.572] | .776 |
| # Wake Bouts | Paternal Abuse | -0.022 [-0.094, 0.058] | .536 | -0.047 [-1.883, 1.814] | .716 |
| Average Sleep Bouts | Maternal Abuse | -0.007 [-0.071, 0.059] | .824 | 0.007 [-0.549, 0.794] | .856 |
| Average Sleep Bouts | Paternal Abuse | -0.022 [-0.091, 0.041] | .520 | -0.058 [-1.548, 2.156] | .652 |

*Note*. W2, wave 2 (2004–2006); W1, wave 1 (1995–1996); W3, wave 3 (2013–2014); B, standardized beta regression; SE, standard error; %, Percentage of; #, Counts of; ACME, average causal mediation effect; CI, confidence interval; ADE, average direct effect; Prop., Proportion. All analyses adjusted for these W1 covariates: panic disorder severity; generalized anxiety disorder severity; major depressive disorder severity; college education status; total household income; and parental psychopathology status.

Table S10

*W2 Active Wake State Actigraphy as a Mediator of W1 Child Abuse Predicting W3 Panic Disorder Severity (N = 308) (Sensitivity Analysis)*

| Mediator Label | Predictor | a Path (β (SE)) | a Path p | b Path (β (SE)) | b Path p |
| --- | --- | --- | --- | --- | --- |
| Total Activity Counts | Maternal Abuse | 6909.393 (SE = 3315.055) | **.038** | 0 (SE = 0) | .693 |
| Total Activity Counts | Paternal Abuse | 1978.307 (SE = 3241.695) | .542 | 0 (SE = 0) | .693 |
| Average Activity Counts/min | Maternal Abuse | 7.123 (SE = 3.327) | **.033** | 0 (SE = 0.001) | .578 |
| Average Activity Counts/min | Paternal Abuse | 2.02 (SE = 3.254) | .535 | 0 (SE = 0.001) | .578 |
| Max Activity Counts | Maternal Abuse | -0.422 (SE = 11.286) | .970 | 0 (SE = 0) | .704 |
| Max Activity Counts | Paternal Abuse | 19.428 (SE = 11.036) | .079 | 0 (SE = 0) | .704 |
| Wake Time | Maternal Abuse | 2.673 (SE = 3.226) | .408 | 0.001 (SE = 0.001) | .374 |
| Wake Time | Paternal Abuse | -1.224 (SE = 3.155) | .698 | 0.001 (SE = 0.001) | .374 |
| % Wake Time | Maternal Abuse | 0.195 (SE = 0.28) | .486 | 0.011 (SE = 0.007) | .109 |
| % Wake Time | Paternal Abuse | -0.11 (SE = 0.274) | .688 | 0.011 (SE = 0.007) | .109 |
| # Wake Bouts | Maternal Abuse | -0.852 (SE = 0.929) | .360 | -0.003 (SE = 0.002) | .110 |
| # Wake Bouts | Paternal Abuse | -0.205 (SE = 0.908) | .821 | -0.003 (SE = 0.002) | .110 |
| Average Sleep Bouts | Maternal Abuse | 0.036 (SE = 0.041) | .380 | 0.082 (SE = 0.045) | .069 |
| Average Sleep Bouts | Paternal Abuse | -0.012 (SE = 0.04) | .768 | 0.082 (SE = 0.045) | .069 |
| Mediator Label | Predictor | ACME [95% CI] | ACME p | ADE [95% CI] | ADE p |
| Total Activity Counts | Maternal Abuse | 0.002 [-0.008, 0.012] | .728 | -0.007 [-0.07, 0.052] | .840 |
| Total Activity Counts | Paternal Abuse | 0.001 [-0.005, 0.007] | .848 | -0.024 [-0.096, 0.043] | .488 |
| Average Activity Counts/min | Maternal Abuse | 0.002 [-0.007, 0.013] | .568 | -0.007 [-0.07, 0.057] | .824 |
| Average Activity Counts/min | Paternal Abuse | 0 [-0.005, 0.007] | .884 | -0.023 [-0.094, 0.044] | .480 |
| Max Activity Counts | Maternal Abuse | 0 [-0.004, 0.005] | .996 | -0.006 [-0.069, 0.051] | .884 |
| Max Activity Counts | Paternal Abuse | -0.001 [-0.009, 0.004] | .772 | -0.021 [-0.095, 0.047] | .540 |
| Wake Time | Maternal Abuse | 0.001 [-0.003, 0.008] | .568 | -0.01 [-0.069, 0.048] | .732 |
| Wake Time | Paternal Abuse | -0.001 [-0.006, 0.003] | .768 | -0.022 [-0.093, 0.052] | .516 |
| % Wake Time | Maternal Abuse | 0.002 [-0.005, 0.01] | .560 | -0.01 [-0.068, 0.05] | .752 |
| % Wake Time | Paternal Abuse | -0.001 [-0.009, 0.005] | .640 | -0.021 [-0.094, 0.048] | .540 |
| # Wake Bouts | Maternal Abuse | 0.003 [-0.006, 0.013] | .528 | -0.009 [-0.075, 0.055] | .764 |
| # Wake Bouts | Paternal Abuse | 0 [-0.008, 0.007] | .944 | -0.022 [-0.099, 0.056] | .540 |
| Average Sleep Bouts | Maternal Abuse | 0.003 [-0.005, 0.014] | .432 | -0.008 [-0.072, 0.048] | .856 |
| Average Sleep Bouts | Paternal Abuse | -0.001 [-0.014, 0.01] | .792 | -0.021 [-0.089, 0.05] | .548 |
| Mediator Label | Predictor | Total Effect [95% CI] | Total Effect p | Prop. Mediated [95% CI] | Prop. Mediated p |
| Total Activity Counts | Maternal Abuse | -0.006 [-0.067, 0.053] | .856 | -0.02 [-2.787, 1.331] | .824 |
| Total Activity Counts | Paternal Abuse | -0.023 [-0.096, 0.046] | .488 | -0.001 [-0.604, 0.66] | .968 |
| Average Activity Counts/min | Maternal Abuse | -0.005 [-0.065, 0.06] | .884 | -0.018 [-1.81, 2.637] | .844 |
| Average Activity Counts/min | Paternal Abuse | -0.022 [-0.094, 0.046] | .504 | 0.001 [-1.13, 0.806] | .956 |
| Max Activity Counts | Maternal Abuse | -0.006 [-0.07, 0.051] | .888 | 0 [-0.492, 0.458] | .996 |
| Max Activity Counts | Paternal Abuse | -0.022 [-0.097, 0.046] | .524 | 0.02 [-0.807, 0.983] | .784 |
| Wake Time | Maternal Abuse | -0.008 [-0.068, 0.05] | .780 | -0.006 [-0.901, 0.962] | .908 |
| Wake Time | Paternal Abuse | -0.023 [-0.095, 0.051] | .512 | 0.007 [-0.344, 0.605] | .800 |
| % Wake Time | Maternal Abuse | -0.008 [-0.068, 0.052] | .828 | -0.004 [-1.293, 2.178] | .948 |
| % Wake Time | Paternal Abuse | -0.023 [-0.097, 0.048] | .520 | 0.024 [-0.715, 1.798] | .736 |
| # Wake Bouts | Maternal Abuse | -0.006 [-0.072, 0.054] | .832 | 0 [-2.456, 2.301] | 1.000 |
| # Wake Bouts | Paternal Abuse | -0.022 [-0.1, 0.056] | .552 | 0.002 [-1.037, 0.969] | .976 |
| Average Sleep Bouts | Maternal Abuse | -0.006 [-0.069, 0.054] | .908 | -0.008 [-2.042, 1.862] | .924 |
| Average Sleep Bouts | Paternal Abuse | -0.022 [-0.09, 0.053] | .504 | 0.02 [-1.165, 3.266] | .792 |

*Note*. W2, wave 2 (2004–2006); W1, wave 1 (1995–1996); W3, wave 3 (2013–2014); B, standardized beta regression; SE, standard error; %, Percentage of; #, Counts of; ACME, average causal mediation effect; CI, confidence interval; ADE, average direct effect; Prop., Proportion. All analyses adjusted for these W1 covariates: panic disorder severity; generalized anxiety disorder severity; major depressive disorder severity; college education status; total household income; and parental psychopathology status.

Table S11

*W2 Resting State Actigraphy as a Mediator of W1 Child Affection Predicting W3 Panic Disorder Severity (N = 308) (Sensitivity Analysis)*

| Mediator Label | Predictor | a Path (β (SE)) | a Path p | b Path (β (SE)) | b Path p |
| --- | --- | --- | --- | --- | --- |
| Total Activity Counts | Maternal Affection | -81.939 (SE = 96.144) | .395 | 0 (SE = 0) | **.037** |
| Total Activity Counts | Paternal Affection | -29.628 (SE = 61.879) | .632 | 0 (SE = 0) | **.037** |
| Average Activity Counts/min | Maternal Affection | -0.179 (SE = 0.2) | .372 | 0.006 (SE = 0.003) | .079 |
| Average Activity Counts/min | Paternal Affection | -0.06 (SE = 0.129) | .639 | 0.006 (SE = 0.003) | .079 |
| Max Activity Counts | Maternal Affection | -0.841 (SE = 2.227) | .706 | 0 (SE = 0) | .321 |
| Max Activity Counts | Paternal Affection | -0.883 (SE = 1.434) | .538 | 0 (SE = 0) | .321 |
| Wake Time | Maternal Affection | -0.055 (SE = 0.356) | .878 | 0.004 (SE = 0.002) | **.043** |
| Wake Time | Paternal Affection | 0.003 (SE = 0.229) | .990 | 0.004 (SE = 0.002) | **.043** |
| % Wake Time | Maternal Affection | -0.023 (SE = 0.073) | .752 | 0.015 (SE = 0.009) | .107 |
| % Wake Time | Paternal Affection | 0.003 (SE = 0.047) | .952 | 0.015 (SE = 0.009) | .107 |
| # Wake Bouts | Maternal Affection | 0.082 (SE = 0.146) | .575 | 0.006 (SE = 0.005) | .179 |
| # Wake Bouts | Paternal Affection | 0.16 (SE = 0.094) | .089 | 0.006 (SE = 0.005) | .179 |
| Average Sleep Bouts | Maternal Affection | 0.023 (SE = 0.072) | .756 | -0.006 (SE = 0.009) | .524 |
| Average Sleep Bouts | Paternal Affection | -0.008 (SE = 0.047) | .861 | -0.006 (SE = 0.009) | .524 |
| Mediator Label | Predictor | ACME [95% CI] | ACME p | ADE [95% CI] | ADE p |
| Total Activity Counts | Maternal Affection | -0.001 [-0.005, 0.001] | .460 | -0.006 [-0.027, 0.016] | .560 |
| Total Activity Counts | Paternal Affection | 0 [-0.002, 0.001] | .476 | 0.004 [-0.013, 0.023] | .716 |
| Average Activity Counts/min | Maternal Affection | -0.001 [-0.004, 0.001] | .452 | -0.006 [-0.028, 0.016] | .636 |
| Average Activity Counts/min | Paternal Affection | 0 [-0.002, 0.001] | .556 | 0.004 [-0.018, 0.024] | .716 |
| Max Activity Counts | Maternal Affection | 0 [-0.003, 0.002] | .828 | -0.007 [-0.029, 0.015] | .504 |
| Max Activity Counts | Paternal Affection | 0 [-0.002, 0.001] | .704 | 0.005 [-0.014, 0.024] | .632 |
| Wake Time | Maternal Affection | 0 [-0.003, 0.003] | .964 | -0.007 [-0.027, 0.015] | .480 |
| Wake Time | Paternal Affection | 0 [-0.002, 0.002] | .944 | 0.003 [-0.017, 0.024] | .716 |
| % Wake Time | Maternal Affection | 0 [-0.004, 0.002] | .744 | -0.007 [-0.03, 0.016] | .524 |
| % Wake Time | Paternal Affection | 0 [-0.001, 0.002] | .936 | 0.003 [-0.019, 0.022] | .744 |
| # Wake Bouts | Maternal Affection | 0.001 [-0.001, 0.003] | .600 | -0.008 [-0.031, 0.013] | .504 |
| # Wake Bouts | Paternal Affection | 0.001 [-0.001, 0.004] | .400 | 0.003 [-0.018, 0.025] | .720 |
| Average Sleep Bouts | Maternal Affection | 0 [-0.001, 0.001] | .768 | -0.008 [-0.032, 0.014] | .440 |
| Average Sleep Bouts | Paternal Affection | 0 [-0.001, 0.001] | .908 | 0.003 [-0.016, 0.022] | .720 |
| Mediator Label | Predictor | Total Effect [95% CI] | Total Effect p | Prop. Mediated [95% CI] | Prop. Mediated p |
| Total Activity Counts | Maternal Affection | -0.007 [-0.028, 0.016] | .480 | 0.046 [-1.083, 1.726] | .660 |
| Total Activity Counts | Paternal Affection | 0.003 [-0.014, 0.022] | .740 | -0.013 [-1.346, 0.829] | .864 |
| Average Activity Counts/min | Maternal Affection | -0.007 [-0.031, 0.015] | .580 | 0.039 [-1.939, 1.315] | .736 |
| Average Activity Counts/min | Paternal Affection | 0.003 [-0.018, 0.023] | .748 | -0.004 [-0.68, 1.115] | .896 |
| Max Activity Counts | Maternal Affection | -0.007 [-0.03, 0.015] | .492 | 0.004 [-0.716, 0.683] | .880 |
| Max Activity Counts | Paternal Affection | 0.005 [-0.014, 0.024] | .648 | -0.004 [-0.619, 0.558] | .880 |
| Wake Time | Maternal Affection | -0.007 [-0.028, 0.016] | .468 | 0.012 [-0.879, 0.997] | .848 |
| Wake Time | Paternal Affection | 0.004 [-0.017, 0.024] | .728 | 0.002 [-0.85, 1.018] | .960 |
| % Wake Time | Maternal Affection | -0.007 [-0.029, 0.016] | .472 | 0.011 [-0.831, 1.308] | .872 |
| % Wake Time | Paternal Affection | 0.003 [-0.02, 0.022] | .748 | 0.003 [-0.755, 0.469] | .924 |
| # Wake Bouts | Maternal Affection | -0.007 [-0.03, 0.014] | .544 | -0.008 [-0.993, 0.974] | .880 |
| # Wake Bouts | Paternal Affection | 0.005 [-0.016, 0.025] | .644 | 0.022 [-2.21, 2.016] | .804 |
| Average Sleep Bouts | Maternal Affection | -0.009 [-0.032, 0.014] | .448 | 0.001 [-0.216, 0.383] | .888 |
| Average Sleep Bouts | Paternal Affection | 0.003 [-0.016, 0.022] | .736 | 0 [-0.655, 0.374] | .972 |

*Note*. W2, wave 2 (2004–2006); W1, wave 1 (1995–1996); W3, wave 3 (2013–2014); B, standardized beta regression; SE, standard error; %, Percentage of; #, Counts of; ACME, average causal mediation effect; CI, confidence interval; ADE, average direct effect; Prop., Proportion. All analyses adjusted for these W1 covariates: panic disorder severity; generalized anxiety disorder severity; major depressive disorder severity; college education status; total household income; and parental psychopathology status.

Table S12

*W2 Sleeping State Actigraphy as a Mediator of W1 Child Affection Predicting W3 Panic Disorder Severity (N = 308) (Sensitivity Analysis)*

| Mediator Label | Predictor | a Path (β (SE)) | a Path p | b Path (β (SE)) | b Path p |
| --- | --- | --- | --- | --- | --- |
| Total Activity Counts | Maternal Affection | -27.193 (SE = 48.448) | .575 | 0 (SE = 0) | .063 |
| Total Activity Counts | Paternal Affection | 8.227 (SE = 31.181) | .792 | 0 (SE = 0) | .063 |
| Average Activity Counts/min | Maternal Affection | -0.064 (SE = 0.113) | .571 | 0.009 (SE = 0.006) | .119 |
| Average Activity Counts/min | Paternal Affection | 0.027 (SE = 0.073) | .708 | 0.009 (SE = 0.006) | .119 |
| Max Activity Counts | Maternal Affection | -0.64 (SE = 1.886) | .734 | 0 (SE = 0) | .446 |
| Max Activity Counts | Paternal Affection | -0.047 (SE = 1.214) | .969 | 0 (SE = 0) | .446 |
| Wake Time | Maternal Affection | -0.016 (SE = 0.224) | .942 | 0.005 (SE = 0.003) | .092 |
| Wake Time | Paternal Affection | 0.087 (SE = 0.144) | .545 | 0.005 (SE = 0.003) | .092 |
| % Wake Time | Maternal Affection | -0.01 (SE = 0.053) | .854 | 0.017 (SE = 0.013) | .173 |
| % Wake Time | Paternal Affection | 0.027 (SE = 0.034) | .435 | 0.017 (SE = 0.013) | .173 |
| # Wake Bouts | Maternal Affection | 0.069 (SE = 0.117) | .560 | 0.008 (SE = 0.006) | .179 |
| # Wake Bouts | Paternal Affection | 0.073 (SE = 0.076) | .338 | 0.008 (SE = 0.006) | .179 |
| Average Sleep Bouts | Maternal Affection | 0.043 (SE = 0.078) | .587 | -0.009 (SE = 0.009) | .304 |
| Average Sleep Bouts | Paternal Affection | -0.029 (SE = 0.05) | .566 | -0.009 (SE = 0.009) | .304 |
| Mediator Label | Predictor | ACME [95% CI] | ACME p | ADE [95% CI] | ADE p |
| Total Activity Counts | Maternal Affection | -0.001 [-0.004, 0.002] | .560 | -0.007 [-0.027, 0.014] | .552 |
| Total Activity Counts | Paternal Affection | 0 [-0.001, 0.002] | .724 | 0.003 [-0.017, 0.022] | .744 |
| Average Activity Counts/min | Maternal Affection | -0.001 [-0.004, 0.002] | .612 | -0.007 [-0.03, 0.014] | .484 |
| Average Activity Counts/min | Paternal Affection | 0 [-0.001, 0.002] | .680 | 0.003 [-0.017, 0.023] | .748 |
| Max Activity Counts | Maternal Affection | 0 [-0.002, 0.001] | .896 | -0.008 [-0.029, 0.014] | .480 |
| Max Activity Counts | Paternal Affection | 0 [-0.001, 0.001] | .956 | 0.004 [-0.016, 0.024] | .700 |
| Wake Time | Maternal Affection | 0 [-0.003, 0.002] | .924 | -0.008 [-0.029, 0.014] | .516 |
| Wake Time | Paternal Affection | 0 [-0.001, 0.002] | .516 | 0.004 [-0.016, 0.024] | .724 |
| % Wake Time | Maternal Affection | 0 [-0.003, 0.002] | .912 | -0.009 [-0.029, 0.012] | .412 |
| % Wake Time | Paternal Affection | 0 [-0.001, 0.002] | .508 | 0.004 [-0.015, 0.023] | .724 |
| # Wake Bouts | Maternal Affection | 0.001 [-0.002, 0.005] | .732 | -0.009 [-0.029, 0.014] | .412 |
| # Wake Bouts | Paternal Affection | 0 [-0.002, 0.003] | .580 | 0.003 [-0.015, 0.025] | .800 |
| Average Sleep Bouts | Maternal Affection | 0 [-0.002, 0.001] | .648 | -0.006 [-0.027, 0.018] | .588 |
| Average Sleep Bouts | Paternal Affection | 0 [-0.001, 0.001] | .536 | 0.004 [-0.017, 0.023] | .676 |
| Mediator Label | Predictor | Total Effect [95% CI] | Total Effect p | Prop. Mediated [95% CI] | Prop. Mediated p |
| Total Activity Counts | Maternal Affection | -0.008 [-0.028, 0.012] | .508 | 0.016 [-2.014, 1.224] | .852 |
| Total Activity Counts | Paternal Affection | 0.004 [-0.016, 0.022] | .728 | 0.004 [-0.819, 0.869] | .924 |
| Average Activity Counts/min | Maternal Affection | -0.008 [-0.03, 0.013] | .456 | 0.017 [-1.194, 1.489] | .804 |
| Average Activity Counts/min | Paternal Affection | 0.004 [-0.017, 0.023] | .732 | 0.002 [-0.914, 0.858] | .948 |
| Max Activity Counts | Maternal Affection | -0.008 [-0.029, 0.014] | .480 | 0.001 [-0.592, 0.588] | .944 |
| Max Activity Counts | Paternal Affection | 0.004 [-0.017, 0.025] | .704 | 0 [-0.432, 0.448] | .988 |
| Wake Time | Maternal Affection | -0.008 [-0.03, 0.014] | .488 | 0.007 [-0.771, 1.276] | .860 |
| Wake Time | Paternal Affection | 0.004 [-0.016, 0.024] | .672 | 0.013 [-1.495, 1.268] | .812 |
| % Wake Time | Maternal Affection | -0.009 [-0.029, 0.012] | .404 | 0.007 [-0.685, 1.084] | .844 |
| % Wake Time | Paternal Affection | 0.004 [-0.014, 0.024] | .664 | 0.007 [-1.264, 1.062] | .884 |
| # Wake Bouts | Maternal Affection | -0.008 [-0.029, 0.016] | .448 | -0.001 [-1.087, 1.066] | .972 |
| # Wake Bouts | Paternal Affection | 0.004 [-0.015, 0.025] | .752 | 0.002 [-0.996, 1.287] | .972 |
| Average Sleep Bouts | Maternal Affection | -0.007 [-0.028, 0.018] | .564 | 0.003 [-0.5, 0.55] | .908 |
| Average Sleep Bouts | Paternal Affection | 0.004 [-0.016, 0.024] | .660 | 0.007 [-0.699, 0.666] | .828 |

*Note*. W2, wave 2 (2004–2006); W1, wave 1 (1995–1996); W3, wave 3 (2013–2014); B, standardized beta regression; SE, standard error; %, Percentage of; #, Counts of; ACME, average causal mediation effect; CI, confidence interval; ADE, average direct effect; Prop., Proportion. All analyses adjusted for these W1 covariates: panic disorder severity; generalized anxiety disorder severity; major depressive disorder severity; college education status; total household income; and parental psychopathology status.

Table S13

*W2 Active Wake State Actigraphy as a Mediator of W1 Child Affection Predicting W3 Panic Disorder Severity (N = 308) (Sensitivity Analysis)*

| Mediator Label | Predictor | a Path (β (SE)) | a Path p | b Path (β (SE)) | b Path p |
| --- | --- | --- | --- | --- | --- |
| Total Activity Counts | Maternal Affection | -2218.146 (SE = 1199.348) | .065 | 0 (SE = 0) | .812 |
| Total Activity Counts | Paternal Affection | -6.099 (SE = 771.909) | .994 | 0 (SE = 0) | .812 |
| Average Activity Counts/min | Maternal Affection | -1.852 (SE = 1.206) | .126 | 0 (SE = 0.001) | .680 |
| Average Activity Counts/min | Paternal Affection | -0.169 (SE = 0.776) | .827 | 0 (SE = 0.001) | .680 |
| Max Activity Counts | Maternal Affection | 1.082 (SE = 4.079) | .791 | 0 (SE = 0) | .672 |
| Max Activity Counts | Paternal Affection | -2.974 (SE = 2.625) | .258 | 0 (SE = 0) | .672 |
| Wake Time | Maternal Affection | -1.164 (SE = 1.161) | .317 | 0 (SE = 0.001) | .396 |
| Wake Time | Paternal Affection | 0.011 (SE = 0.747) | .989 | 0 (SE = 0.001) | .396 |
| % Wake Time | Maternal Affection | -0.022 (SE = 0.101) | .825 | 0.011 (SE = 0.007) | .108 |
| % Wake Time | Paternal Affection | -0.031 (SE = 0.065) | .635 | 0.011 (SE = 0.007) | .108 |
| # Wake Bouts | Maternal Affection | 0.278 (SE = 0.335) | .407 | -0.003 (SE = 0.002) | .129 |
| # Wake Bouts | Paternal Affection | -0.073 (SE = 0.215) | .734 | -0.003 (SE = 0.002) | .129 |
| Average Sleep Bouts | Maternal Affection | -0.021 (SE = 0.015) | .145 | 0.079 (SE = 0.045) | .084 |
| Average Sleep Bouts | Paternal Affection | 0.012 (SE = 0.009) | .199 | 0.079 (SE = 0.045) | .084 |
| Mediator Label | Predictor | ACME [95% CI] | ACME p | ADE [95% CI] | ADE p |
| Total Activity Counts | Maternal Affection | 0 [-0.004, 0.003] | .816 | -0.007 [-0.028, 0.015] | .480 |
| Total Activity Counts | Paternal Affection | 0 [-0.001, 0.001] | .972 | 0.004 [-0.014, 0.022] | .672 |
| Average Activity Counts/min | Maternal Affection | 0 [-0.003, 0.002] | .644 | -0.008 [-0.029, 0.014] | .512 |
| Average Activity Counts/min | Paternal Affection | 0 [-0.001, 0.001] | .996 | 0.005 [-0.014, 0.025] | .684 |
| Max Activity Counts | Maternal Affection | 0 [-0.001, 0.001] | .964 | -0.008 [-0.03, 0.015] | .524 |
| Max Activity Counts | Paternal Affection | 0 [-0.001, 0.002] | .708 | 0.004 [-0.014, 0.024] | .708 |
| Wake Time | Maternal Affection | -0.001 [-0.003, 0.001] | .616 | -0.007 [-0.031, 0.016] | .584 |
| Wake Time | Paternal Affection | 0 [-0.001, 0.001] | .996 | 0.004 [-0.015, 0.024] | .744 |
| % Wake Time | Maternal Affection | 0 [-0.003, 0.002] | .844 | -0.008 [-0.032, 0.017] | .520 |
| % Wake Time | Paternal Affection | 0 [-0.002, 0.001] | .712 | 0.003 [-0.016, 0.022] | .728 |
| # Wake Bouts | Maternal Affection | -0.001 [-0.004, 0.001] | .368 | -0.007 [-0.03, 0.014] | .576 |
| # Wake Bouts | Paternal Affection | 0 [-0.001, 0.002] | .848 | 0.003 [-0.015, 0.023] | .784 |
| Average Sleep Bouts | Maternal Affection | -0.002 [-0.009, 0.003] | .492 | -0.006 [-0.028, 0.013] | .580 |
| Average Sleep Bouts | Paternal Affection | 0.001 [-0.001, 0.004] | .420 | 0.003 [-0.018, 0.026] | .768 |
| Mediator Label | Predictor | Total Effect [95% CI] | Total Effect p | Prop. Mediated [95% CI] | Prop. Mediated p |
| Total Activity Counts | Maternal Affection | -0.007 [-0.029, 0.015] | .464 | 0.022 [-0.692, 0.878] | .784 |
| Total Activity Counts | Paternal Affection | 0.004 [-0.014, 0.022] | .672 | 0.002 [-0.352, 0.742] | .924 |
| Average Activity Counts/min | Maternal Affection | -0.008 [-0.028, 0.015] | .480 | 0.023 [-1.17, 0.825] | .756 |
| Average Activity Counts/min | Paternal Affection | 0.005 [-0.014, 0.025] | .688 | 0.001 [-0.332, 0.553] | .956 |
| Max Activity Counts | Maternal Affection | -0.008 [-0.031, 0.014] | .528 | 0 [-0.28, 0.581] | .988 |
| Max Activity Counts | Paternal Affection | 0.004 [-0.014, 0.024] | .696 | 0.003 [-0.867, 1.083] | .884 |
| Wake Time | Maternal Affection | -0.007 [-0.031, 0.016] | .544 | 0.016 [-0.772, 1.128] | .760 |
| Wake Time | Paternal Affection | 0.004 [-0.015, 0.024] | .744 | 0.001 [-1.074, 0.694] | .964 |
| % Wake Time | Maternal Affection | -0.008 [-0.033, 0.018] | .520 | 0.016 [-0.716, 0.78] | .764 |
| % Wake Time | Paternal Affection | 0.003 [-0.016, 0.022] | .740 | 0.001 [-0.949, 1.218] | .980 |
| # Wake Bouts | Maternal Affection | -0.008 [-0.031, 0.013] | .532 | 0.031 [-0.941, 1.426] | .692 |
| # Wake Bouts | Paternal Affection | 0.003 [-0.015, 0.024] | .752 | 0.012 [-0.73, 1.362] | .864 |
| Average Sleep Bouts | Maternal Affection | -0.008 [-0.03, 0.012] | .488 | 0.093 [-1.715, 2.161] | .652 |
| Average Sleep Bouts | Paternal Affection | 0.004 [-0.016, 0.026] | .684 | 0.024 [-1.561, 1.922] | .840 |

*Note*. W2, wave 2 (2004–2006); W1, wave 1 (1995–1996); W3, wave 3 (2013–2014); B, standardized beta regression; SE, standard error; %, Percentage of; #, Counts of; ACME, average causal mediation effect; CI, confidence interval; ADE, average direct effect; Prop., Proportion. All analyses adjusted for these W1 covariates: panic disorder severity; generalized anxiety disorder severity; major depressive disorder severity; college education status; total household income; and parental psychopathology status.

Table S14

*ρ-Based Sensitivity Analyses for Unmeasured Confounding for Analyses of Maternal and Paternal Abuse As Predictors*

| Mediator | Predictor | ACME at ρ = 0 | ρ* (ACME = 0) | \|ρ*\| | *R²* (Mediator Model) | *R²* (Outcome Model) | Robustness Classification |
| --- | --- | --- | --- | --- | --- | --- | --- |
| **Actigraphy Rest Stage** |  |  |  |  |  |  |  |
| # Wake Bouts | Maternal Abuse | -0.0018 | 0.1370 | 0.1370 | 6.29% | 21.56% | Modest (0.10 <= \|ρ*\| < 0.20) |
| # Wake Bouts | Paternal Abuse | 0.0089 | 0.1370 | 0.1370 | 6.29% | 21.56% | Modest (0.10 <= \|ρ*\| < 0.20) |
| % Wake Time | Maternal Abuse | 0.0007 | 0.2090 | 0.2090 | 12.78% | 23.55% | Moderate (0.20 <= \|ρ*\| < 0.40) |
| % Wake Time | Paternal Abuse | 0.0160 | 0.2090 | 0.2090 | 12.78% | 23.55% | Moderate (0.20 <= \|ρ*\| < 0.40) |
| Mean Activity Counts/min | Maternal Abuse | 0.0047 | 0.2160 | 0.2160 | 13.93% | 23.77% | Moderate (0.20 <= \|ρ*\| < 0.40) |
| Mean Activity Counts/min | Paternal Abuse | 0.0156 | 0.2160 | 0.2160 | 13.93% | 23.77% | Moderate (0.20 <= \|ρ*\| < 0.40) |
| Mean Sleep Bouts | Maternal Abuse | -0.0006 | -0.0530 | 0.0530 | 2.29% | 20.28% | Weak (\|ρ*\| < 0.10) |
| Mean Sleep Bouts | Paternal Abuse | 0.0026 | -0.0530 | 0.0530 | 2.29% | 20.28% | Weak (\|ρ*\| < 0.10) |
| **Actigraphy Sleep Stage** |  |  |  |  |  |  |  |
| # Wake Bouts | Maternal Abuse | -0.0014 | 0.1210 | 0.1210 | 4.91% | 21.22% | Modest (0.10 <= \|ρ*\| < 0.20) |
| # Wake Bouts | Paternal Abuse | 0.0077 | 0.1210 | 0.1210 | 4.91% | 21.22% | Modest (0.10 <= \|ρ*\| < 0.20) |
| % Wake Time | Maternal Abuse | 0.0005 | 0.1940 | 0.1940 | 11.25% | 23.07% | Modest (0.10 <= \|ρ*\| < 0.20) |
| % Wake Time | Paternal Abuse | 0.0141 | 0.1940 | 0.1940 | 11.25% | 23.07% | Modest (0.10 <= \|ρ*\| < 0.20) |
| Mean Activity Counts/min | Maternal Abuse | 0.0023 | 0.2140 | 0.2140 | 13.74% | 23.71% | Moderate (0.20 <= \|ρ*\| < 0.40) |
| Mean Activity Counts/min | Paternal Abuse | 0.0160 | 0.2140 | 0.2140 | 13.74% | 23.71% | Moderate (0.20 <= \|ρ*\| < 0.40) |
| Mean Sleep Bouts | Maternal Abuse | -0.0002 | -0.0610 | 0.0610 | 2.08% | 20.35% | Weak (\|ρ*\| < 0.10) |
| Mean Sleep Bouts | Paternal Abuse | 0.0030 | -0.0610 | 0.0610 | 2.08% | 20.35% | Weak (\|ρ*\| < 0.10) |
| Wake Time Length | Maternal Abuse | 0.0007 | 0.1990 | 0.1990 | 12.29% | 23.21% | Modest (0.10 <= \|ρ*\| < 0.20) |
| Wake Time Length | Paternal Abuse | 0.0150 | 0.1990 | 0.1990 | 12.29% | 23.21% | Modest (0.10 <= \|ρ*\| < 0.20) |

*Note.* ACME, average causal mediation effect. This sensitivity test could not be conducted in some models with significant ACME estimates in the primary mediation models due to non-convergence issues.

Table S15

*ρ-Based Sensitivity Analyses for Unmeasured Confounding for Analyses of Maternal and Paternal Affection As Predictors*

| Mediator | Predictor | ACME at ρ = 0 | ρ* (ACME = 0) | \|ρ*\| | *R²* (Mediator Model) | *R²* (Outcome Model) | Robustness Classification |
| --- | --- | --- | --- | --- | --- | --- | --- |
| **Actigraphy Rest Stage** |  |  |  |  |  |  |  |
| # Wake Bouts | Maternal Affection | -0.0005 | 0.1350 | 0.1350 | 5.63% | 21.72% | Modest (0.10 <= \|rho*\| < 0.20) |
| # Wake Bouts | Paternal Affection | 0.0015 | 0.1350 | 0.1350 | 5.63% | 21.72% | Modest (0.10 <= \|rho*\| < 0.20) |
| % Wake Time | Maternal Affection | -0.0020 | 0.2080 | 0.2080 | 11.03% | 23.73% | Moderate (0.20 <= \|rho*\| < 0.40) |
| % Wake Time | Paternal Affection | 0.0017 | 0.2080 | 0.2080 | 11.03% | 23.73% | Moderate (0.20 <= \|rho*\| < 0.40) |
| Mean Activity Counts/min | Maternal Affection | -0.0027 | 0.2160 | 0.2160 | 11.89% | 23.99% | Moderate (0.20 <= \|rho*\| < 0.40) |
| Mean Activity Counts/min | Paternal Affection | 0.0014 | 0.2160 | 0.2160 | 11.89% | 23.99% | Moderate (0.20 <= \|rho*\| < 0.40) |
| Mean Sleep Bouts | Maternal Affection | -0.0004 | -0.0520 | 0.0520 | 1.59% | 20.48% | Weak (\|rho*\| < 0.10) |
| Mean Sleep Bouts | Paternal Affection | 0.0002 | -0.0520 | 0.0520 | 1.59% | 20.48% | Weak (\|rho*\| < 0.10) |
| **Actigraphy Sleep Stage** |  |  |  |  |  |  |  |
| # Wake Bouts | Maternal Affection | -0.0006 | 0.1200 | 0.1200 | 3.91% | 21.41% | Modest (0.10 <= \|rho*\| < 0.20) |
| # Wake Bouts | Paternal Affection | 0.0010 | 0.1200 | 0.1200 | 3.91% | 21.41% | Modest (0.10 <= \|rho*\| < 0.20) |
| % Wake Time | Maternal Affection | -0.0019 | 0.1930 | 0.1930 | 10.00% | 23.23% | Modest (0.10 <= \|rho*\| < 0.20) |
| % Wake Time | Paternal Affection | 0.0020 | 0.1930 | 0.1930 | 10.00% | 23.23% | Modest (0.10 <= \|rho*\| < 0.20) |
| Mean Activity Counts/min | Maternal Affection | -0.0025 | 0.2130 | 0.2130 | 12.14% | 23.87% | Moderate (0.20 <= \|rho*\| < 0.40) |
| Mean Activity Counts/min | Paternal Affection | 0.0020 | 0.2130 | 0.2130 | 12.14% | 23.87% | Moderate (0.20 <= \|rho*\| < 0.40) |
| Mean Sleep Bouts | Maternal Affection | -0.0006 | -0.0600 | 0.0600 | 1.44% | 20.55% | Weak (\|rho*\| < 0.10) |
| Mean Sleep Bouts | Paternal Affection | 0.0003 | -0.0600 | 0.0600 | 1.44% | 20.55% | Weak (\|rho*\| < 0.10) |

*Note.* ACME, average causal mediation effect. This sensitivity test could not be conducted in some models with significant ACME estimates in the primary mediation models due to non-convergence issues.

References

Lakens, D. (2013). Calculating and reporting effect sizes to facilitate cumulative science: a practical primer for t-tests and ANOVAs. *Frontiers in Psychology, 4*. doi:10.3389/fpsyg.2013.00863

Rosenthal, R. (1994). *Parametric measures of effect size*. New York: Russell Sage Foundation.

Simes, R. J. (1986). An improved Bonferroni procedure for multiple tests of significance. *Biometrika, 73*, 751-754. doi:10.2307/2336545
